# ARIBA: rapid antimicrobial resistance genotyping directly from sequencing reads

**DOI:** 10.1099/mgen.0.000131

**Published:** 2017-09-04

**Authors:** Martin Hunt, Alison E Mather, Leonor Sánchez-Busó, Andrew J Page, Julian Parkhill, Jacqueline A Keane, Simon R Harris

**Affiliations:** ^1^​ Infection Genomics, Wellcome Trust Sanger Institute, Wellcome Genome Campus, Hinxton, CB10 1SA, UK; ^2^​ Department of Veterinary Medicine, University of Cambridge, Madingley Road, Cambridge, CB3 0ES, UK

**Keywords:** antimicrobial resistance, whole genome sequencing, genotyping, bacteria, sequence typing

## Abstract

Antimicrobial resistance (AMR) is one of the major threats to human and animal health worldwide, yet few high-throughput tools exist to analyse and predict the resistance of a bacterial isolate from sequencing data. Here we present a new tool, ARIBA, that identifies AMR-associated genes and single nucleotide polymorphisms directly from short reads, and generates detailed and customizable output. The accuracy and advantages of ARIBA over other tools are demonstrated on three datasets from Gram-positive and Gram-negative bacteria, with ARIBA outperforming existing methods.

## Abbreviations

AMR, antimicrobial resistance; ARIBA, Antimicrobial Resistance Identification By Assembly; AZM, azithromycin; MIC, minimum inhibitory concentration; MLST, multi-locus sequence type; SNP, single nucleotide polymorphism; VRE, vancomycin-resistant enterococci; VSE, vancomycin-susceptible enterococci.

## Data Summary

1. The ARIBA software is open source and available for Linux at Github under the GNU GPLv3 licence (url – https://github.com/sanger-pathogens/ariba).

2. Accession numbers for all sequencing reads used are provided in the supplementary material.

## Impact Statement

Antimicrobial resistance (AMR) is a growing threat to global public health and is an issue in need of urgent action. Despite increased research into AMR and availability of genomic sequencing data, few bioinformatics tools exist to identify AMR genes and/or variants directly from sequencing reads. Current tools are limited by the accuracy and completeness of analysis they can perform, are specific to certain reference data or species, or are only available via low-throughput web servers. We introduce ARIBA, a new software tool for identifying AMR determinants from sequence reads, which overcomes the drawbacks associated with current tools. It is applicable to generic reference data, in addition to supporting the public databases ARG-ANNOT, CARD, MEGAres and ResFinder. ARIBA is fast, computationally efficient and more accurate than the other available command line tools at identifying the presence of acquired AMR genes and variants and verifying their completeness and predicted functionality. ARIBA output is highly customizable and can be compared with phenotypic resistance data using an integrated plotting function or interactively interrogated using the Phandango visualization tool.

## Introduction

Antimicrobial-resistant infections have become one of the leading threats to human health, with a conservative estimate of 700 000 directly attributed deaths per year worldwide [1]. If we do not address this threat, this figure is estimated to rise to 10 million by 2050 [1]. An important component of any strategy to tackle antimicrobial resistance (AMR) is having rapid and accurate methods for identifying markers of resistance. Our understanding of the mechanisms and diversity of AMR is improving, in part due to the increased availability of genome sequence data, with the use of genome sequencing in personalized medicine set to become one key tool in the fight against AMR. However, there are currently few bioinformatics tools that can identify AMR determinants directly from the data produced by widely-used sequencing technologies. The methods that are available are limited in the types of AMR mechanisms they can detect and/or are not scalable to high-throughput environments.

Limitations of existing tools include being available only via web services that are not high-throughput; being restricted to a specific set of reference sequences which may not exhaustively represent current knowledge of AMR for all microbial species; requiring assembled genome sequences as input; an inability to identify and interpret single-nucleotide-polymorphism (SNP)-based AMR determinants; and having high computational resource requirements. Most tools fall into one of two categories: those that align sequencing reads to a set of reference genes, and those that search for reference gene matches in *de novo* assembled sequences. The widely-used SRST2 [2] is an example of a method based on aligning reads to a set of reference sequences in order to predict the presence of those genes in a sample. KmerResistance [3] employs a similar approach, but uses *k*-mer matching between sequencing reads and reference genes to identify gene presence. Although SRST2 and KmerResistance can be used with custom reference gene sets, they cannot directly identify or interpret variants, such as SNPs that confer resistance, and so are limited to identifying resistance that is conferred by the presence of a gene, or a particular pre-defined allele of a gene. Mykrobe predictor [4] is an extremely fast tool that matches *k*-mers in reads to a reference graph, and although it can identify variants, it is currently limited to *Staphylococcus aureus* and *Mycobacterium tuberculosis*, and it is not possible for users to provide their own databases of AMR determinants with which to interrogate their data.

The majority of other AMR detection tools require assembled sequences as input, which are computationally expensive to generate from reads, and assembly errors or failures caused by the complexity of assembling complete genomes *de novo* can lead to AMR determinants being missed. For these reasons, alignment-based approaches have previously been shown to be superior to the use of *de novo* assembled sequences [2, 3] for AMR gene detection. Tools that use assembled sequences as input include ResFinder [5], ARG-ANNOT [6], SSTAR [7] and RAST [8]. These methods match assembled sequences to reference genes, usually using the blast [9] algorithm, in order to identify AMR genes.

Here we present a new tool, called ARIBA (Antimicrobial Resistance Identification By Assembly), that uses a combined mapping/alignment and targeted local assembly approach to identify AMR genes and variants efficiently and accurately from paired sequencing reads. Using targeted local assembly considerably reduces the complexity of the assembly process, while providing contiguous gene or nucleotide sequences without the ambiguity of the interpretation of aligned data. ARIBA can easily be provided with custom reference sequence-sets, and includes support for a number of public databases: ARG-ANNOT [6], CARD [10], MEGARes [11] and ResFinder [5]. It distinguishes between sequences that are coding or non-coding, and provides details on each sequence present in the sample. It verifies whether or not identified genes are complete, truncated or fragmented in the sample, and reports SNPs and indels within sequences with interpretations of their effect, such as frameshifts, non-synonymous changes or nonsense mutations. To facilitate easier interpretations of results, ARIBA includes functions to summarize results for multiple samples. These summaries are compatible with the Phandango interactive visualization tool [12]. If minimum inhibitory concentration (MIC) data are available for samples, ARIBA allows statistical analysis and plotting of MIC against genotype. Beyond AMR, ARIBA can be used more generally to find any input sequences of interest. It provides inbuilt support for the PlasmidFinder [13] and VFDB [14] databases, and functionality for multi-locus sequence typing (MLST) using data from PubMLST [15].

## Methods

### ARIBA

**Fig. 1.**
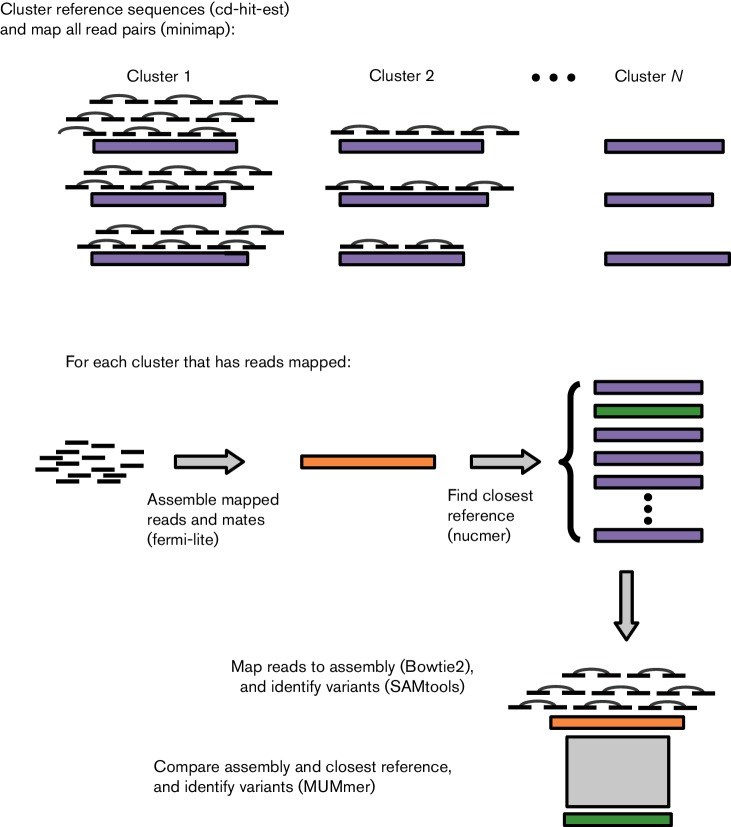
Overview of the ARIBA mapping and targeted assembly pipeline.

We developed ARIBA to identify AMR determinants from public or custom databases using paired read data as input. Fig. 1 provides an overview of the approach. Briefly, reference sequences in the AMR database are clustered by similarity using CD-HIT [16]. Reads are mapped to the reference sequences using minimap [17] to produce a set of reads for each cluster. These reads map to at least one of the sequences in that cluster. The reads for each cluster and their sequence pairs are assembled independently using fermi-lite (https://github.com/lh3/fermi-lite) under a variety of parameter combinations, and the closest reference sequence to the resulting contigs is identified with the program nucmer from the MUMmer package [18]. The assembly is compared to the reference sequence to identify completeness and any variants between the sequences using the nucmer and show-snps programs from MUMmer. The reads for the cluster are mapped to the assembly with Bowtie2 [19] and variants are called with SAMtools [20]. Finally, a detailed report is made of all the sequences identified in the sample, including, but not limited to, the presence or absence of variants pre-defined to be of importance to AMR.

### Obtaining input data

ARIBA requires reference sequences and, optionally, information about SNPs known to confer resistance. ARIBA supports several public resources, allowing the user to download the data easily and convert it into a form for use with the pipeline. ARG-ANNOT, CARD, PlasmidFinder, ResFinder, VFDB, and the SRST2 version of ARG-ANNOT are currently available. These can be obtained by running the command

ariba getref name_of_resource output_directory

Alternatively, reference data can be provided by the user. Reference sequences can be coding or non-coding. Coding sequences are subjected to extra checks for consistency, as described below, and extra analysis is performed on them, such as determining if SNPs are synonymous or nonsynonymous. Further, each reference sequence is classified as ‘presence/absence’ where the existence of a sequence within a sample confers antimicrobial resistance, or ‘variant only’ where a known variant is required for antimicrobial resistance.

### Preparing input data

All reference data are checked for consistency, and any sequences or variants that do not pass all checks are completely removed. It is important that the user provides valid input data and checks ARIBA log files for removed data. Any coding sequences are required to begin with a start codon, be a complete open reading frame, and end with a stop codon. All reading frames are checked on both strands and any sequence that fails any of the requirements is removed. Any ‘known variant’ defined in the input file is required to match the reference sequence. For example, if a known variant for a reference is I42L, then the translated reference sequence must have an ‘I’ or ‘L’ at amino acid 42 or the variant will be removed. The remaining sequences are clustered using the cd-hit-est program from CD-HIT, using the options –c 0.9 (minimum 90 % sequence identity) –s 0 (no length difference cutoff). These defaults can be changed by the user. Since any sequence can be coding or non-coding, and presence/absence or variant only, four disjoint sets of sequences are created. These sets of sequences are kept separate, with each one clustered individually. The input data are prepared by running the command

ariba prepareref -f sequences.fasta -m metadata.tsv prepareref.out

where the reference sequences are in the FASTA file sequences.fasta and extra information, such as variants, is in the tab-delimited file metadata.tsv. These input files are generated automatically when running ariba getref.

### Cluster analysis

Once the reference data are prepared, the main ARIBA pipeline can be run using paired reads and the reference data as input. The command is

ariba run prepareref.out reads_1.fq reads_2.fq run.out

where the directory prepareref.out was created with ariba prepareref. First, the reads are mapped to all of the input sequences (that passed quality filters), using minimap with a *k*-mer length, *k*, of 15 and minimizer window size of 10. A read is considered to be mapped by minimap if: 1) the match length is at least 50 or half of the read length, whichever is smaller; 2) the start position of the match is within 1.1 *k* of the start of the read or the reference sequence; 3) the same as for 2), but for the end position of the match. The result is that reads that match completely to the centre of the reference sequence, and reads that overhang the ends of the reference sequence are counted as mapped. The situation is illustrated in Fig. S1 (available in the online Supplementary Material) and explained in detail in the Supplementary Material.

Any read that maps, or whose mate maps, to a reference sequence is allocated to the cluster to which the reference sequence belongs. Note that the same read can be allocated to more than one cluster, for example if two reference sequences lie next to each other in the genome. Each resulting cluster has a set of reference sequences, as determined by CD-HIT, and a set of paired reads.

Each cluster is processed independently as follows (see Fig. S2). To reduce assembly running time, the reads input to the assembler are randomly downsampled to a maximum of 50× coverage by default. Since the true reference sequence for this cluster is not yet known, the coverage is (over)estimated using the length of the longest reference sequence for the cluster. The reads are assembled using fermi-lite, which is run using the options -l x -c y,10 000 where x takes the values 6, 15 and y takes the values 4, 17, 30, resulting in six distinct assemblies. These parameters were chosen because they were found to have the greatest effect on assembly quality, with no benefits outside those ranges.

The assemblies are compared against all reference sequences from the cluster using nucmer. The best within-cluster nucmer match is identified by maximizing for the percent of the reference sequence that is assembled. Ties are broken by taking the highest percent identity, the largest value of -lfrom minimap, and finally the largest value of -c from minimap. Next, the contig subsequence from the best nucmer match is compared against all reference sequences (across all clusters). The best match is chosen using the same criteria as for the within-cluster best match, and the corresponding reference sequence is chosen to be the closest reference sequence for this cluster. If the closest reference sequence does not belong to the cluster, then no further analysis is performed and the cluster is not counted as present.

Next, the assembly is compared to the closest reference sequence using the MUMmer suite of programs. The contigs are aligned to the reference sequence using nucmer, then SNPS and indels are identified between the sequences using show-snps. This information is used to determine the overall success of the assembly, encoded into a bitwise flag (i.e. a single integer). For example, the reference sequence could have a complete match to a single contig. In the case where the reference sequence is a gene, the matching position in the contig is checked for any nonsense mutations. A complete explanation of the flag and the various scenarios it encodes is given in the Supplementary Material. The meaning of a flag N can be determined using the command

ariba flag N

which will report a breakdown of the flag N.

All reads from the cluster are mapped to the contigs using Bowtie2 and the read depth at each contig position and SNPs are identified using SAMtools mpileup. Finally, the alignment and variant information is used to generate a summary for this sample, which includes the success of the assembly, whether or not the sample has SNPs of interest and the read depth at those SNPs.

The output of ariba run includes a report file containing the summary information of each cluster, plus FASTA files of the assemblies and detailed logging information.

### Summarizing results

The results of multiple runs of ARIBA across different samples can be summarized by running

ariba summary out report.*.tsv

where report.*.tsv is a list of reports, each made with a call to ariba run (Fig. S3). This command generates input files to Phandango, and a CSV file that can be easily viewed in spreadsheet applications. A key output for each sample and cluster is an interpretation of the flag, which summarises the matches to the reference sequence as one of: no, partial, fragmented, interrupted, yes_nonunique, or yes (Fig. S4).

Since Phandango requires a tree, ARIBA determines a clustering tree using the contents of its CSV file, which means that it is generated from the calls involving the reference genes and SNPs of interest. The distance between two samples is defined as the number of columns in the CSV file that agree, and an UPGMA tree is generated from the distance matrix using DendroPy [21]. Users may wish to provide their own tree, calculated using sequence-based methods.

### Benchmarking

The performance of ARIBA was evaluated on three datasets, to illustrate all aspects of its functionality and to benchmark against other available methods. For these comparisons, we focussed on command line tools that can use custom reference data, specifically SRST2 and KmerResistance.

ARIBA version 2.8.1 was used, together with dependencies Bowtie2 2.2.29, CD-HIT 4.6, MUMmer 3.1 and Python packages dendropy 4.2.0, pyfastaq 3.15.0 (https://github.com/sanger-pathogens/Fastaq), pymummer 0.10.2 (https://github.com/sanger-pathogens/pymummer) and pysam 0.10.0 (https://github.com/pysam-developers/pysam). We used KmerResistance checkout 041bc89b832cf6a3b7629d76b4dffb4c7428caab, and SRST2 0.2.0 with the recommended versions Bowtie2 2.1.0 and SAMtools 0.1.18. All software was run with the default settings on the Cloud Infrastructure for Microbial Bioinformatics [22]. The complete terminal commands used are in the Supplementary Material.

###   *Enterococcus faecium*

The first dataset comprises 41 isolates of the Gram-positive bacterium *Enterococcus faecium*, for which the phenotypic resistance to vancomycin is known for each sample [23] (Table S1). This dataset, which was used to evaluate SRST2 in its initial publication [2 ], allowed validation of the accuracy of ARIBA when identifying the presence or absence of genes of interest in each sample, testing the sensitivity of the methods at varying depths of read coverage, and verifying MLST calling by ARIBA and SRST2.

The ARG-ANNOT sequences included with SRST2 were used as reference sequences for the benchmarking on this dataset. However, the VanS-B gene, called ‘47__VanS-B_Gly__VanS-B__1672 no;yes;VanS-B;Gly;AY655721;731–2073;1343’ by SRST2, originally from ARG-ANNOT, was missing its final nucleotide A. This was confirmed by comparing with the GenBank record AY655721. It would cause ARIBA to exclude this sequence because the translation into amino acids results in a sequence that does not end with a stop codon. Therefore an ‘A’ was manually added to the end of the sequence before running ARIBA.

In order to sample the *E. faecium* reads at a range of depths, the reads were mapped to the reference genome CP006620 using Bowtie2 version 2.2.29 with the option -fast-local. The depth for each sample was estimated across the *vanB* gene CP006620.1476 by running SAMtools depth with the options -a -r CP006620 : 774 918–775 946 and calculating the resulting mean depth. This was used as an estimate for read depth and the reads were randomly sampled accordingly (this is implemented in the supplementary script make_read_subsets.pl) using fastaq to_random_subset with a different random seed for each run, producing independent read subsets.

###  *Shigella sonnei*

The second dataset, published by Holt *et al*. [24], consists of 130 globally distributed genomes of *Shigella sonnei* (Table S2), a Gram-negative bacterium that is a causative agent of dysentery. It enabled a comparison of ARIBA, SRST2, and KmerResistance with the manual method employed in the study of Holt *et al*. [24], confirming the accuracy of ARIBA for identifying known resistance SNPs as well as the presence or absence of genes of interest.

The phenotypic resistance profile for a number of antimicrobials is known for each isolate, and is attributable to both acquired resistance genes and SNPs. The three tools were run on all 130 samples using the reference database from CARD, version 1.1.2. To ensure our results were comparable with those originally reported in Table S1 of Holt *et al*. [24], we manually added those AMR genes listed on page 4 of their supplementary text not already included in the database (Table S3). The AMR determinants originally reported in the study of Holt *et al*. [24] were identified from mapping data, and reported as the proportion of bases in the gene sequence that were covered by reads from each isolate. From these originally reported data, we used a cut-off of >90 % to indicate that a gene was present by their method.

In order to interpret the output of each tool as an AMR call, the following rules were used, where all relevant genes are listed in Table S4. A gene was counted as present by ARIBA if ariba summary reported yes or yes_nonunique; present by KmerResistance if it appeared in its output file; and present by SRST2 if it was reported without a ‘?’.

The focus for the genes of interest for each AMR call were those originally identified and reported in Holt *et al.* [24]. Given that the discovery and classification of AMR gene variants is an ongoing process, an AMR gene was called as present if it was either the originally identified gene in Holt *et al.* [24], or in the same CD-HIT cluster. Genes conferring resistance to antimicrobials not examined in the original paper were excluded, as were genes conferring resistance to the antimicrobials examined in the paper but falling in different CD-HIT clusters from the originally identified genes. For each antimicrobial examined, an AMR call for a resistant genotype was identified using the following rules. Ampicillin (Amp): the presence of any gene from a set of *bla*TEM, *bla*CTX-M and *bla*OXA genes. Chloramphenicol (Cmp): the presence of any gene from a set of *cat* genes. Nalidixic acid (Nal): the *gyrA* gene present, together with one of the SNPs S83L, D87G, or D87Y. Streptomycin (Str): both of the *strA* and *strB* genes, or one of the *aadA* genes. Sulfonamides (Sul): any gene from the set of *sul1* and *sul2* genes. Tetracycline (Tet): both of *tetA* +*tetR*, or all of *tetA*, *C*, *D*, *R*, where each of the two sets of *tetA* and *tetR* genes are disjoint. Trimethoprim (Tmp): any one of a set of *dfrA* or *dhfr* genes.

###  *Neisseria gonorrhoeae*

The third dataset comprises data from the sexually-transmitted pathogen *Neisseria gonorrhoeae*, and was used to showcase functionality of ARIBA that is not available in other tools. The data are from five recent studies [25–29] (Table S5), totalling 1517 samples, which include phenotypic data on resistance to four antimicrobials.

First, we created a custom database of gonococcal AMR determinants (Table S6). Unique alleles for each gene from the 2016 World Health Organization gonococcus reference collection [30] and five available *Neisseria meningitidis* complete genomes (H44 - GCA_000191445.1; MC58 - GCA_000008805.1; M01-240149 - GCA_000191465.1; FAM18 - GCA_000009465.1; Z2491 - GCA_000009105.1) were included in the database to allow identification of recombinant genes.

The input files and commands run to create the *N. gonorrhoeae* ARIBA resistance database can be found in the supplementary material. Briefly, for each gene all unique alleles from the reference set were saved in multifasta files. For variant-based resistances, alignments were created in Seaview [31, 32] by translating to amino acid sequences, aligning with Clustal [31] using default parameters and back translating to nucleotides. For each alignment, the aln2meta function of ARIBA was used to produce the files required as input to prepareref. These were combined, along with the sequence files for presence/absence resistance gene files and prepareref run to create the ARIBA database.

To create a phylogenetic tree of all isolates, sequencing reads were aligned to the chromosome of *N. gonorrhoeae* FA1090 (GenBank accession number NC_002946) using BWA MEM (version 0.7.12-r1039) [33] with the options to output alignments for unpaired reads and to mark shorter split hits as secondary. Optical duplicates were removed and indels realigned using GATK [34] MarkDuplicates (version 1.127) and indelRealigner (version 3.4–46), respectively, under their default settings. Variant sites were identified from each isolate using SAMtools (version 1.2) [20] mpileup with options to report DP and DP4 statistics, count orphans, adjust the mapping quality to 50 and increase the maximum depth to 1000, including for indel calling, followed by bcftools (version 1.2) call using a prior of 0.001, a ploidy of 1 and with the option to keep all alternate alleles at variant sites. All sites were further filtered as described previously [35] to produce a multiple sequence alignment. Repeats and prophages in the FA1090 genome were masked from the alignment before variable sites were identified with snp_sites [36] and a neighbour-joining phylogenetic tree created with RapidNJ [37]. Interactive visualization of the phylogenetic tree and ARIBA summary data was carried out in Phandango v0.8.5.

## Results

###  *Enterococcus faecium*

First, we used ARIBA and SRST2 to identify the sequence type of each sample, using the *E. faecium* MLST scheme [38] downloaded from PubMLST. Given that MLST loci are chosen to be conserved, single-copy housekeeping genes, identification of MLST should be a simple test for any gene-detection method. As expected, we found that the results generated by both tools were in complete agreement with the known sequence types provided in Howden *et al*. [23] (Table S1). However, the running time of ARIBA was approximately one-fifth that of SRST2 (Table S7). See Page *et al.* [39] for an in-depth comparison of current MLST tools, including SRST2 and ARIBA.

Seventeen of the samples are known to have VanB-mediated resistance to vancomycin (i.e. are vancomycin-resistant enterococci, VRE) and the remaining 24 samples are known to be susceptible (i.e. are vancomycin-susceptible enterococci, VSE). The phenotypic resistance is due to the presence of an operon comprising up to seven genes: *vanB*, *vanH*, *vanR*, *vanS*, *vanW*, *vanX* and *vanY* [40]. However, *vanW* and *vanY* are not required for resistance [40, 41]. ARIBA, KmerResistance and SRST2 were evaluated using the antimicrobial resistance reference set of genes from SRST2, which is based on ARG-ANNOT and includes all seven genes of interest. All three tools made identical calls on the 17 VRE samples in the *vanB*, *vanH*, *vanR*, *vanS* and *vanX* genes, except for the choice of closest reference sequence in sample SRR980582, which differed for the *vanB* gene (Table S1). Several of the VSE samples contain low-level contamination with *VanA-B* sequences [2]; here, in most cases only ARIBA flagged the genes as partially present at a low read depth, and SRST2 and KmerResistance did not make any prediction about the presence of these genes (Table S1).

The remaining differences between the tools were in the identification of *vanW* and *vanY* in the VRE samples. The discrepancies demonstrate the benefits of the detailed output of ARIBA, when compared to the other tools. A complete description of the differences between the output of the three tools is given in the Supplementary Material (Section 3.2 and Fig. S7). For example, in sample SRR980557, SRST2 reported that the *vanW* gene was present but with one SNP (‘1snp’ in the output), and KmerResistance also reported the gene as present. ARIBA reported a SNP, but provided the further information that it was a nonsense mutation and therefore the gene is likely to be non-functional in that sample.

**Fig. 2.**
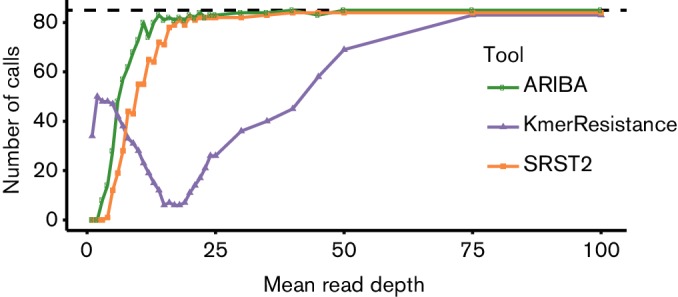
Effect of read depth on the number of gene calls for all five *van* genes in the 17 vancomycin-resistant *E. faecium* samples.

The effect of read depth was assessed on the 17 VRE samples by uniformly sampling from the reads at depths ranging from 1 to 100× coverage of the vancomycin resistance operon. The total number of calls for the five required resistance genes made by each tool across all 17 samples is shown in Fig. 2, and a per-gene breakdown is given in Fig. S5. KmerResistance appears to be optimized for coverage below 5×, and its ability to call the presence of genes decreases in the range 2×–18× before recovering in the range 50–75×. The ability of ARIBA and SRST2 to identify genes improves with read depth, with ARIBA marginally outperforming SRST2. When partial matches to genes are included, ARIBA and SRST2 become more sensitive at lower coverages and ARIBA becomes more sensitive than KmerResistance (Fig. S6).

###  *Shigella sonnei*

**Fig. 3.**
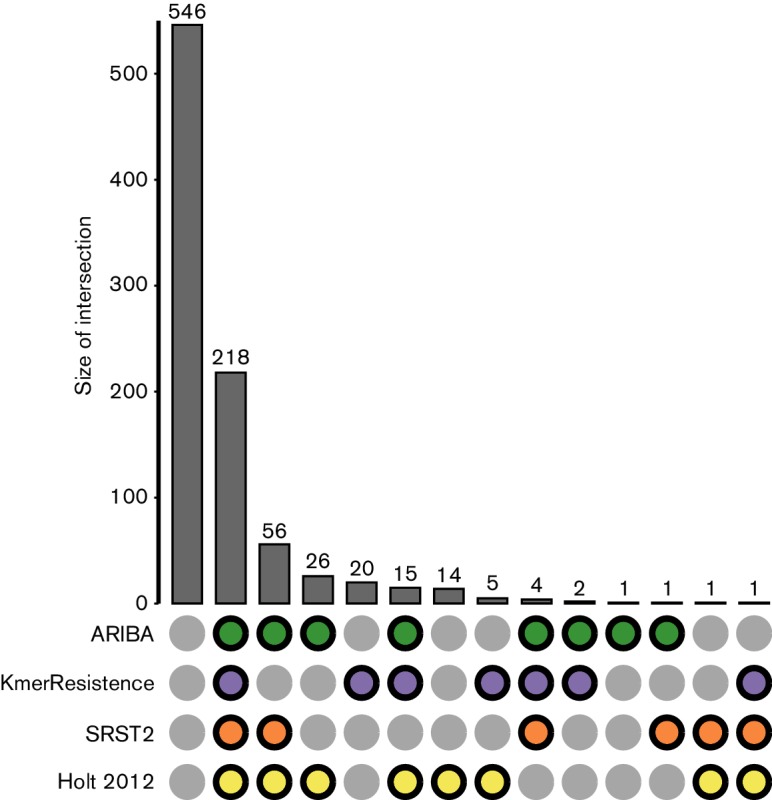
Concordance between AMR calling methods on the *S. sonnei* data. A coloured dot indicates which methods were in agreement. For example, column 3 shows that there were 56 samples where ARIBA, SRST2 and Holt *et al*. [24] (Holt 2012) were in agreement in predicting the presence or absence of AMR. The first column illustrates where no resistance mechanisms were predicted.

With seven antimicrobials and 130 isolates, there was a potential for 910 AMR calls (identification of a gene, set of genes, or SNP). In 546 cases, no calls were made by any method (Table S2). 364 AMR calls were made by at least one of the four methods; 60 % (218/364) were found by all four methods (Fig. 3 and Table S2). Overall, this results in an agreement between the four methods of 84 %: (218 calls in agreement +546 non-calls in agreement)/(910 potential AMR calls).

For the 146 calls where there were discrepancies between the methods, we observed some general trends explaining most of the discordance. First, neither KmerResistance nor SRST2 identify specific SNPs conferring resistance, whereas this is possible with ARIBA and the method of Holt *et al.* [24]. SNPs in the *gyrA* gene, which cause resistance to quinolone antimicrobials, were found by both ARIBA and the manual method of Holt *et al.* in 22 isolates. Second, there were 20 cases where a resistance gene was called by KmerResistance, but not by any other method. Upon further investigation, KmerResistance reported these genes at a low coverage (1.4–4.9×). Third, although KmerResistance appears to be the best at detecting genes present at very low coverage, it made fewer calls of genes at higher coverage than ARIBA and SRST2 (Fig. S8). For example, in isolate ERR028689 *dfrA1* is found at 31× coverage by ARIBA and at 37× by SRST2, but is not reported by KmerResistance. When partial matches are allowed by SRST2 and ARIBA, there are no calls made only by KmerResistance (Fig. S9). However, less stringency could result in false-positive calls. A full report of the calls made by each method for each antimicrobial and isolate examined is in Table S8.

There were only two cases where ARIBA did not match any other method. These involved either differences in identifying SNPs, or a large insertion into an AMR gene. In the first, ARIBA differed from the results reported in Holt *et al*. [24] for samples ERR028676 and ERR028677 when identifying SNPs in the *gyrA* gene that confer resistance to quinolone drugs. ARIBA was confirmed to be correctly reporting the SNPs in each sample by analysing the mapped reads, as described in Supplementary Material.

The second case relates to streptomycin resistance, one mechanism for which requires the presence of both the *strA* and *strB* genes. Sample ERR024606 has an insertion into the AMR gene *strA*, which renders it non-functional. The *strA* gene was called as present by SRST2 with high confidence and a depth of 150×, and at 179× by KmerResistance, and at 100 % coverage by the method of Holt *et al.* [24]. However, ARIBA correctly characterized *strA* as not functionally present as it did not assemble into a single contig; this was manually confirmed to be due to the insertion of *dfrA14* into the middle of *strA* (Fig. S10), similar to that described previously [42]. We found a second instance of an insertion disrupting an AMR gene, in this case *strB* (Fig. S11) in isolate ERR028673, and again ARIBA made the correct call. We note that KmerResistance also made the correct AMR call for streptomycin for this isolate, but only because although it called *strB*, it did not call *strA* (called at 80× and 101× by ARIBA and SRST2, respectively).

###  *Neisseria gonorrhoeae*

The sexually-transmitted pathogen *N. gonorrhoeae* is under strict public health surveillance because isolates resistant to the first-line antimicrobials, azithromycin (AZM) and the extended spectrum cephalosporins (ESCs; i.e. cefixime and ceftriaxone) have been reported worldwide. Here, we illustrate some of the extended features of ARIBA, including the creation and use of customized AMR databases, identification of resistance mutations (SNPs and deletions) in coding and non-coding regions and identification of heterozygous resistance mutations in multicopy rRNAs. We note that this example is intended to be for illustrative purposes only, not an in-depth analysis of gonococcal AMR determinants.

For the purposes of this example, we concentrate on AZM resistance and associated mutations in the 23S rRNA and the *mtrR* gene, which encodes a repressor to the mtr (multiple transferable resistance) efflux system. Our database includes two 23S mutations, A2045G (A2059G *Escherichia coli* numbering) and C2597T (C2611T *Escherichia coli* numbering), which are linked to high-level [43] and low-level [44] AZM resistance, respectively. For *mtrR*, both a G45D substitution and interruption of the gene have been linked to increased efflux leading to reduced susceptibility to multiple antimicrobials.

Visualization of the ARIBA results in Phandango allows patterns of the presence and absence of variants to be viewed against a tree of isolates. ARIBA can create a dendrogram of isolates based on the identified resistance variants, so that when visualized in Phandango, isolates are clustered by shared resistance-determinant profiles. Alternatively, a phylogenetic tree based on SNPs in the core genome of the isolates can be provided to Phandango, as in Fig. S12, making it possible to visualize interactively the distribution of resistance mechanisms across the pathogen population. Based on the occurrences of 23S and *mtrR* variants on independent branches within the phylogenetic tree, it is clear that the variants in our database have emerged multiple times in the gonococcal population. 23S-mediated resistance, in particular, has often emerged but failed to spread, suggesting it may be associated with a fitness cost.

**Fig. 4.**
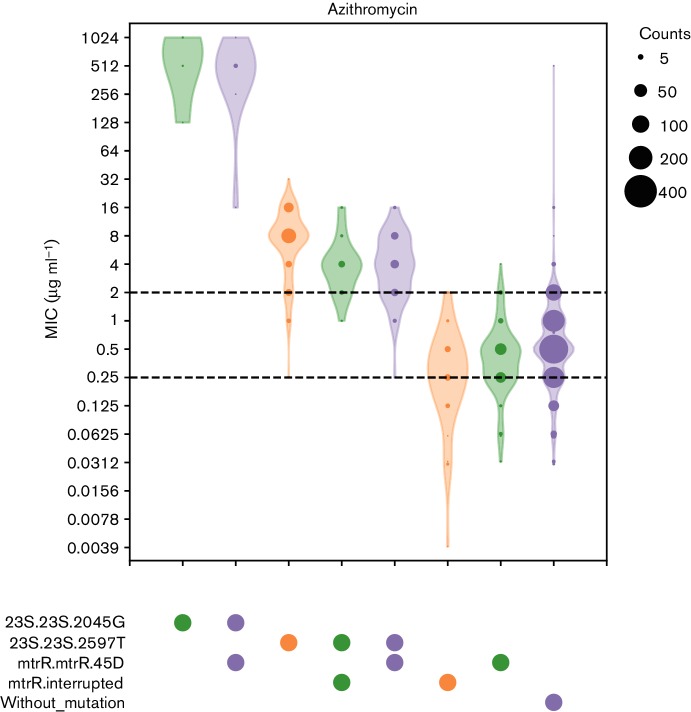
Distribution of MICs (represented on a logarithmic scale) for AZM for all observed combinations of relevant AMR determinants in our custom database. Dotted horizontal lines mark clinical breakpoints. The lower line marks the lowest EUCAST (http://www.eucast.org/clinical_breakpoints/) breakpoint (0.25 µg ml^−1^) and the upper line marks the post-2005 breakpoint used in the USA (2 µg ml^−1^) [46].

Next, we explored the distribution of the minimum inhibitory concentration (MIC) for AZM in isolates with all AZM-related genetic resistance determinants as identified from our database (Fig. S13) using the ‘micplot’ function of ARIBA. This function outputs publication-quality images along with pairwise Mann-Whitney U Test *P*-values and effect sizes. Although, as expected, the 23S mutations in our database show clear evidence of association with resistance, the results for the *mtrR* variants are less clear-cut, being found in both resistant and sensitive isolates. Visualizing MICs of combinations of resistance determinants allows improved understanding of causal versus linked AMR determinants, and of combinations of determinants which may produce a cumulative effect. By default, ARIBA micplot draws all observed combinations of variants found by ARIBA against user-provided MIC data, so that linked and combinatorial determinants are easier to identify. Fig. 4 shows that when separated from linked 23S mutations, the 45D substitution or interruption of *mtrR* alone showed no increase in MIC relative to isolates without a proposed resistance determinant, consistent with other studies [25].

**Fig. 5.**
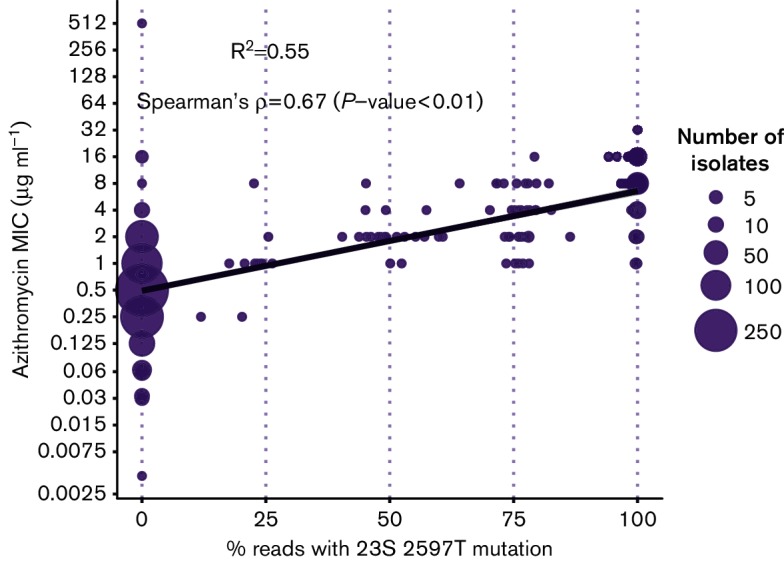
Correlation between the number of alleles containing the 23S C2597T mutation (C2611T in *Escherichia coli*) in AZM-resistant isolates and their MIC values for this antimicrobial.

Although most of the isolates with the 23S mutations exhibited MICs above the 2 µg ml^−1^ breakpoint, some would be identified as susceptible if this breakpoint was strictly applied. *N. gonorrhoeae* usually carries four copies of the ribosomal operon. The C2597T mutation can occur in any number of the 23S copies, with increasing number of copies of the mutated allele having been previously associated with increasing MIC [28, 45]. ARIBA allows the detection of such heterozygous mutations, which can be important for understanding genotype–phenotype relationships. Fig. S14 shows how excluding isolates for which the 23S mutations are heterozygous alters the plots in Fig. 4, reducing the number of isolates falling below the 2 µg ml^−1^ breakpoint. Fig. 5 shows the percentage of reads (used here as a proxy for the number of gene copies) carrying the mutation, as reported by ARIBA, and its correlation with AZM MIC, confirming that increasing copies of the mutation are correlated with increased phenotypic resistance in this dataset. We should note that other AZM resistance mechanisms were not taken into account in this analysis, which is intended for illustrating the features of ARIBA rather than as a detailed analysis of 23S mutations.

## Discussion

Increasing antimicrobial resistance threatens to produce untreatable infections, with catastrophic consequences for public health. While new antimicrobials must be developed, we also need to use our current antimicrobials effectively, using those that are appropriate for the resistances and sensitivities of the infection to be treated. One approach to this will be to use rapid genomics-based approaches to predict resistance, and this in turn will rely on fast, accurate and automatable software tools. Here we have developed and implemented a new tool, ARIBA, that not only outperforms existing tools at identifying AMR genes, but also identifies and classifies variants involved in AMR. In addition to supporting common AMR databases, ARIBA was developed to be easily applied with any input reference data. This means that it could be used to identify any sequences of interest, not just those involved in AMR. The use of local assemblies means that ARIBA can determine effectively whether or not an isolate possesses a copy of a gene that is functional or non-functional, unlike other tools, which do not perform this depth of analysis. Further, as showcased on the *N. gonorrhoeae* data, ARIBA reports the presence of variants, interprets their consequences, and identifies the presence of a variant that is known to cause AMR.

ARIBA is only as good as the quality of the input reference database, and these databases will need to be independently validated, especially if they are intended for clinical use. We note the method assumes that a sample has one gene per reference cluster produced by CD-HIT, which can in rare cases cause undesired results. For example, only one match within a cluster can be chosen, even if there is really more than one sequence from that cluster in a sample. For this reason, we do not recommend using ARIBA with metagenomic data. The method is applicable to paired sequencing reads of high enough quality to produce accurate local assemblies, including, but not limited to Illumina. Future work would be required to adapt the method to use new long-read technologies such as Oxford Nanopore or Pacific Biosciences, however this would require the use of different assembly methods.

In conclusion, we have developed a new tool, ARIBA, that identifies AMR determinants directly from paired sequencing reads, and have demonstrated a number of ways in which it improves upon existing tools: 1) verifies completeness of acquired resistance genes; 2) identifies known causative resistance SNPs; 3) allows exploration of the association of AMR determinants with user-provided MIC data; 4) identifies SNP frequency in multicopy genes, which has been traditionally difficult to resolve due to the complexities of *de novo* assembly; and 5) generally requires less time and computational resources. Thus, the novel approach of mapping followed by targeted assembly of each reference sequence is fast, efficient and accurate when compared to current methods. Moreover, ARIBA reports significantly more details than existing tools, particularly variant calls, enabling a deeper understanding of the resistance associated with each isolate.

## Data bibliography

1. Ballard SA *et al.* GenBank AY655721 (2005).
2. Lam MM *et al.* GenBank CP006620 (2013).
3. Hostetler J *et al.* GenBank GCA_000191445.1 (2010).
4. Tettelin H *et al.* GenBank GCA_000008805.1 (2000).
5. Budroni S *et al.* GenBank GCA_000191465.1 (2010).
6. Bentley SD *et al.* GenBank GCA_000009465.1 (2007).
7. Parkhill J *et al.* GenBank GCA_000009105.1 (2000).

## Supplementary Data

156 Supplementary File 1

## Supplementary Data

2209 Supplementary File 2
