# Supplementary material for: ARIBA: rapid antimicrobial resistance genotyping directly from sequencing reads
Source: Microb Genom. 2017 Sep 4;3(10):e000131. doi: 10.1099/mgen.0.000131 (PMC5695208; doi:10.1099/mgen.0.000131)
Supplement: Supplementary File 2 [file mgen-3-131-s002.pdf]

Supplementary Material of:

ARIBA: Rapid antimicrobial resistance genotyping directly from sequencing reads

Martin Hunt[1], Alison E. Mather[1,2], Leonor Sánchez-Busó[1], Andrew J. Page[1], Julian Parkhill[1], Jacqueline A. Keane[1], Simon R. Harris[1]

[1]Wellcome Trust Sanger Institute, Wellcome Trust Genome Campus, Cambridge, CB10 1SA, UK
[2]Department of Veterinary Medicine, University of Cambridge, Madingley Road, Cambridge, UK, CB3 0ES

Contents

1 Analysis reproducibility **3**

2 ARIBA pipeline **3**

3 *E. faecium* **9**

4 *S. sonnei* **15**

5 *N. gonorrhoeae* **21**

6 Run time and memory **24**

1 Analysis reproducibility

Accession numbers for the raw reads are listed in Supplementary Tables S1, S2, and S5. All other files and scripts used for this publication are included in the GitHub repository `https://github.com/martinghunt/ariba-publication`. This includes the reference files used as input to ARIBA, KmerResistance, and SRST2, and how to generate them. It also includes the output files from each tool (except for the BAM and pileup files made by SRST2, in order to reduce space), and scripts used to generate figures 2–5, supplementary figures S5, S6, S8, S9, S13–S15, tables, and this PDF file. We note that Figure 4 was automatically generated by ARIBA, then the lower left labels were tidied manually for publication. Further, a Docker file is included so that the analysis can be easily reproduced. Each script referred to below in this text can be found in the `Scripts/` directory of the GitHub repository.

2 ARIBA pipeline

The first stage of the ARIBA pipeline is to map all read pairs to all reference sequences, in order to produce a set of reads for each cluster of reference sequences. Reads that map with their entire length, or overhang the end of a reference sequence, are counted as mapped providing the match is "good enough", using the following criteria. Minimap mapping coordinates are approximate because each read is placed but is not aligned. Therefore some tolerance is allowed at the end of each reported mapping. We allow $1.1k$ unaligned nucleotides at each end of a read, where k is the k-mer length used by minimap (by default $k = 15$).

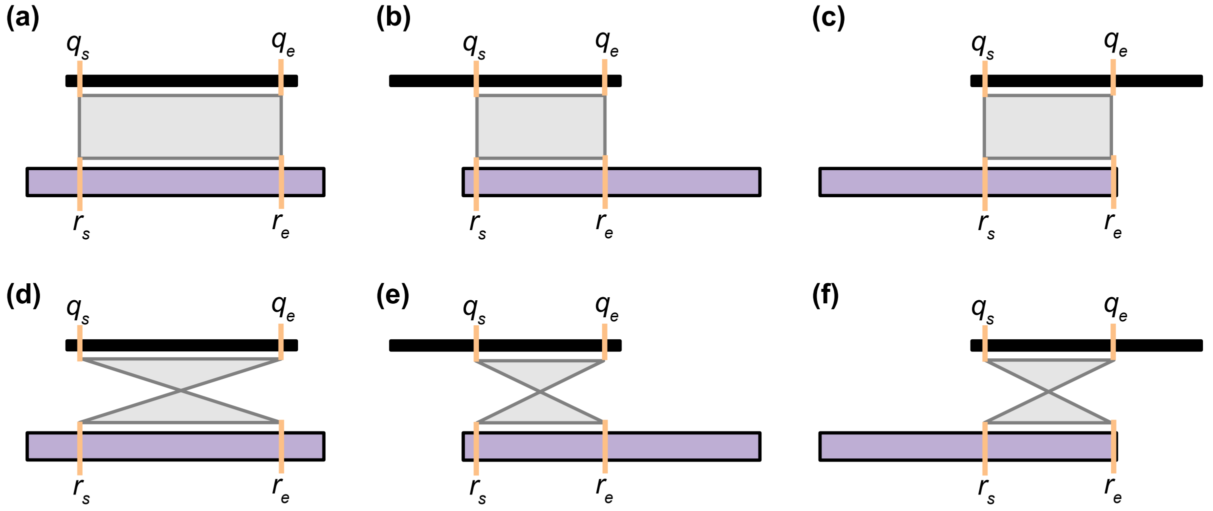

Figure S1: Visualisation of criteria used to determine whether or not a read (in black) was considered to be mapped to the reference (in purple) by minimap. q_s and q_e are the start and end positions in the read of the minimap match. r_s and r_e are the start and end positions in the reference of the minimap match. See text for details.

Let q_ℓ and r_ℓ be the length of the read and reference sequences respectively, q_s and q_e the match start and end positions in the read, r_s and r_e the the match start and end positions in the reference, and $\varepsilon = 1.1k$. The possible orientations are shown in S1. Assume that $q_s < q_e$ and $r_s < r_e$ (by reverse-complementing the read if necessary). The first requirement is that enough of the read matches, specifically that

$$q_e - q_s \geqslant \min(50, q_\ell/2).$$

If the read is mapped in the same orientation as the reference (cases (a),(b),(c) in Supplementary Figure S1), then we require

$$q_s < \varepsilon \quad \text{or} \quad r_s < \varepsilon$$

and

$$q_\ell - q_e < \varepsilon \quad \text{or} \quad r_\ell - r_e < \varepsilon$$

to count the read as mapped. On the other hand, if the read is mapped onto the reverse strand of the reference (cases (d),(e),(f) in Supplementary Figure S1), then we require

$$q_s < \varepsilon \quad \text{or} \quad r_\ell - r_e < \varepsilon$$

and

$$q_\ell - q_e < \varepsilon \quad \text{or} \quad r_s < \varepsilon$$

to count the read as mapped.

If either read of a pair is counted mapped to a given reference sequence, then that read pair is allocated to the cluster to which that reference sequence belongs. Since for each read all mappings reported by minimap are considered, a read pair can be allocated to more than one cluster.

Each cluster is handled using the methods described in the main text, and outlined in Supplementary Figure S2. A variety of situations can arise for each sample. The possibilities are encoded in a bitwise flag, where each possibility is set to 'true' or 'false', with the following meanings.

- **assembled:** the assembly is compared to the reference sequence using nucmer. If at least 95% of the reference sequence has nucmer matches to the assembly, then `assembled` is true. The 95% is a default value that can be changed with the command line option `--assembled_threshold`. Note that this says nothing about how many contigs represent the gene (see the next option `assembled_into_one_contig`).
- **assembled_into_one_contig:** this is set to true if `assembled` is true, and also there is a single contig with a nucmer match that covers at least 95% of the reference sequence. Note that there could still be other contigs that match the reference (see `region_assembled_twice`).
- **region_assembled_twice:** this is set to true if more than 3% of the reference sequence has more than one match to the assembly. The 3% cutoff can be changed with the command line option `--unique_threshold`.
- **complete_gene:** if there is a match to the full length of the reference sequence, or if the match is not quite complete, then ARIBA will try to extend it to the nearest start and stop codons. If this is successful, and the only stop codon is at the end of the inferred gene sequence, then `complete_gene` is set to true. This will never be set if the reference is a non-coding sequence.
- **unique_contig:** this is set to true if there is exactly one contig in the assembly that has nucmer matches to the reference sequence.
- **scaffold_graph_bad:** the reads are mapped back to the assembly and links between the contigs from read pair information is used to construct a scaffolding graph. If there is any ambiguity in this graph, for example the end of contig A could join to the start of contig B or contig C, then `scaffold_graph_bad` is set to true.

- **assembly_fail:** this is set when the assembler produces no output. The most likely cause is a few reads spuriously mapped to the reference sequence, whose depth is too low to assemble.
- **variants_suggest_collapsed_repeat:** after mapping the reads back to the assembly, variants are called using SAMtools. If SAMtools calls any variants in any position that matches to the reference gene, then this is set to true. It suggests that the assembly has collapsed more than one sequence down into one sequence, hence the reads suggesting variants. Alternatively, this could be caused by a mixed input sample.
- **hit_both_strands:** this means there is a contig that has two (or more) matches to the reference, but the matches are in opposite orientations.
- **has_variant:** this is set to true if there is any variant between the assembly and the reference. For a noncoding sequence, this means any nucleotide change. For a gene, this means any non-synonymous change. Except that a known variant is only counted when the assembly has the variant type, as opposed to the wild type (bear in mind that the reference could have the wild type or the variant type).
- **ref_seq_choose_fail:** this is set to true if something went wrong when trying to find the closest reference sequence within a cluster.

ARIBA includes a utility to explain the meaning of a flag. For example, running

```
ariba flag 27
```

results in the following output

```
Meaning of flag 27
[X] assembled
[X] assembled_into_one_contig
[ ] region_assembled_twice
[X] complete_gene
[X] unique_contig
[ ] scaffold_graph_bad
[ ] assembly_fail
[ ] variants_suggest_collapsed_repeat
[ ] hit_both_strands
[ ] has_variant
[ ] ref_seq_choose_fail
```

where an `X` means that part of the flag is true. In this case, the assembly consisted of one unique contig that included the complete gene sequence.

Runs across multiple samples can be summarised using the `summary` function of ARIBA, as outlined in Supplementary Figure S3. A key column of the output from summary is the ‘assembled’ column, which reports, for each sample and each gene, the status of the ARIBA assembly. The method used to calculate this column is shown in Supplementary Figure S4.

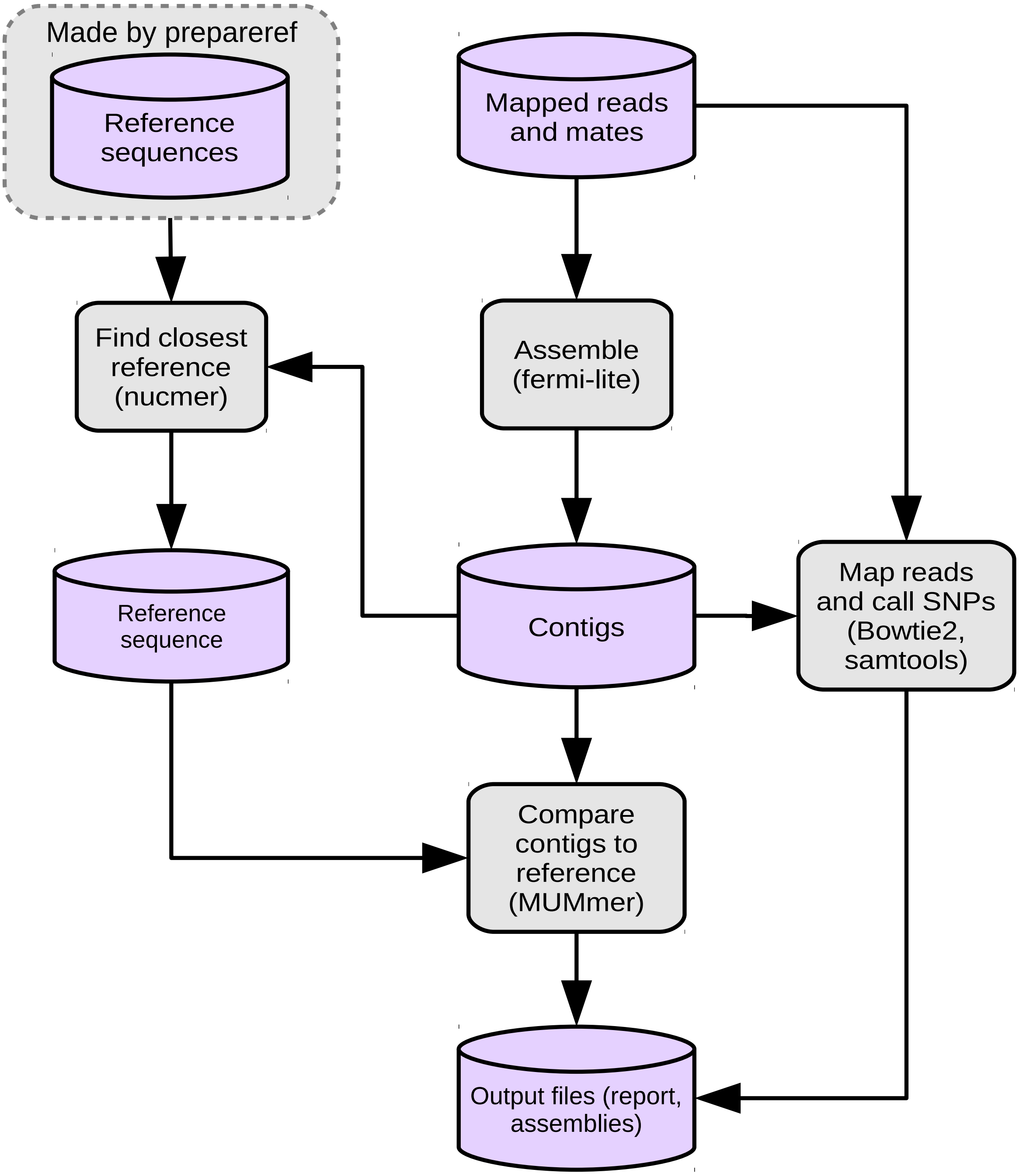

Figure S2: Cluster processing methods (performed on each cluster during `ariba run`)

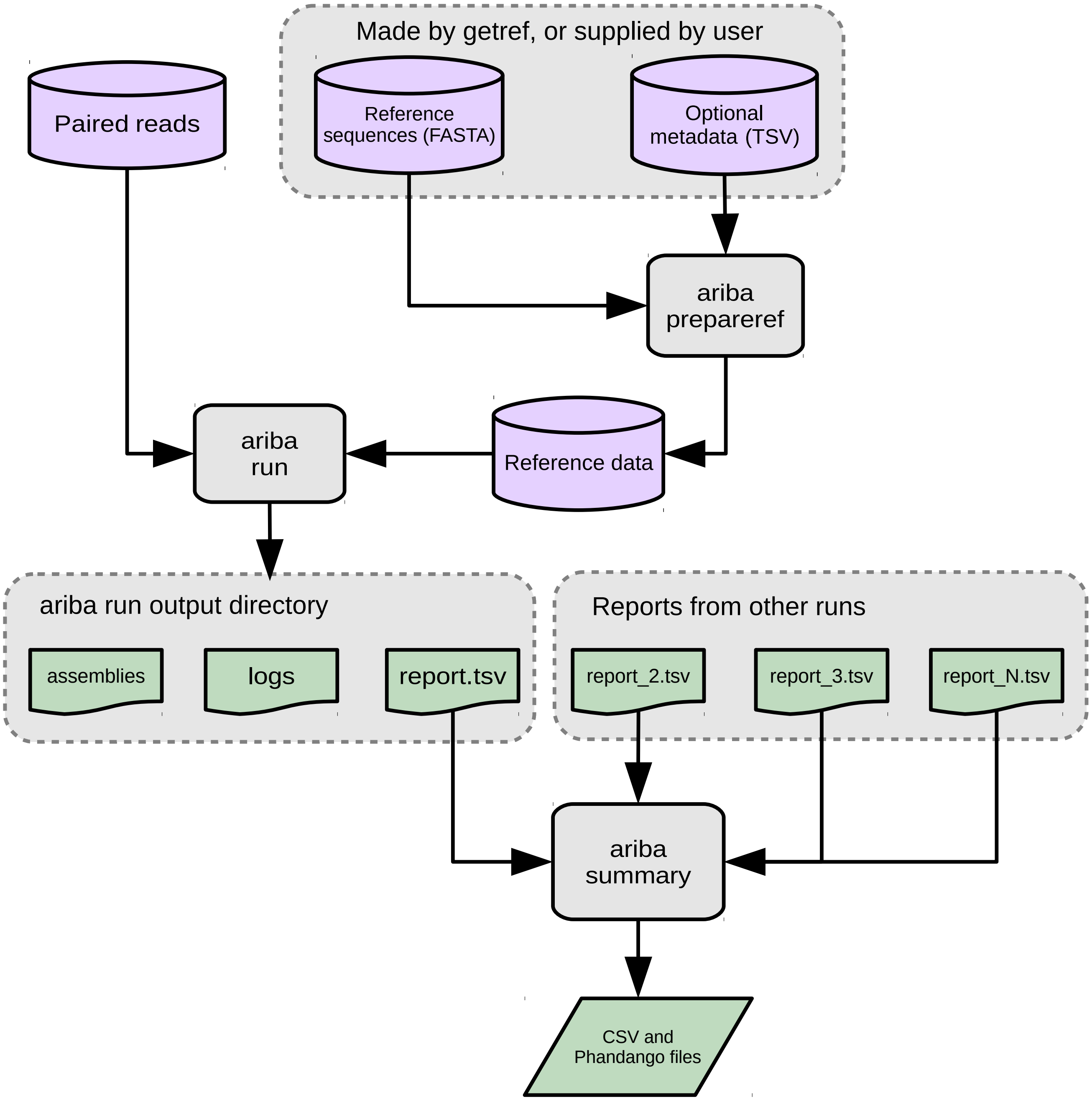

Figure S3: Overview of ARIBA methods, when run on multiple samples

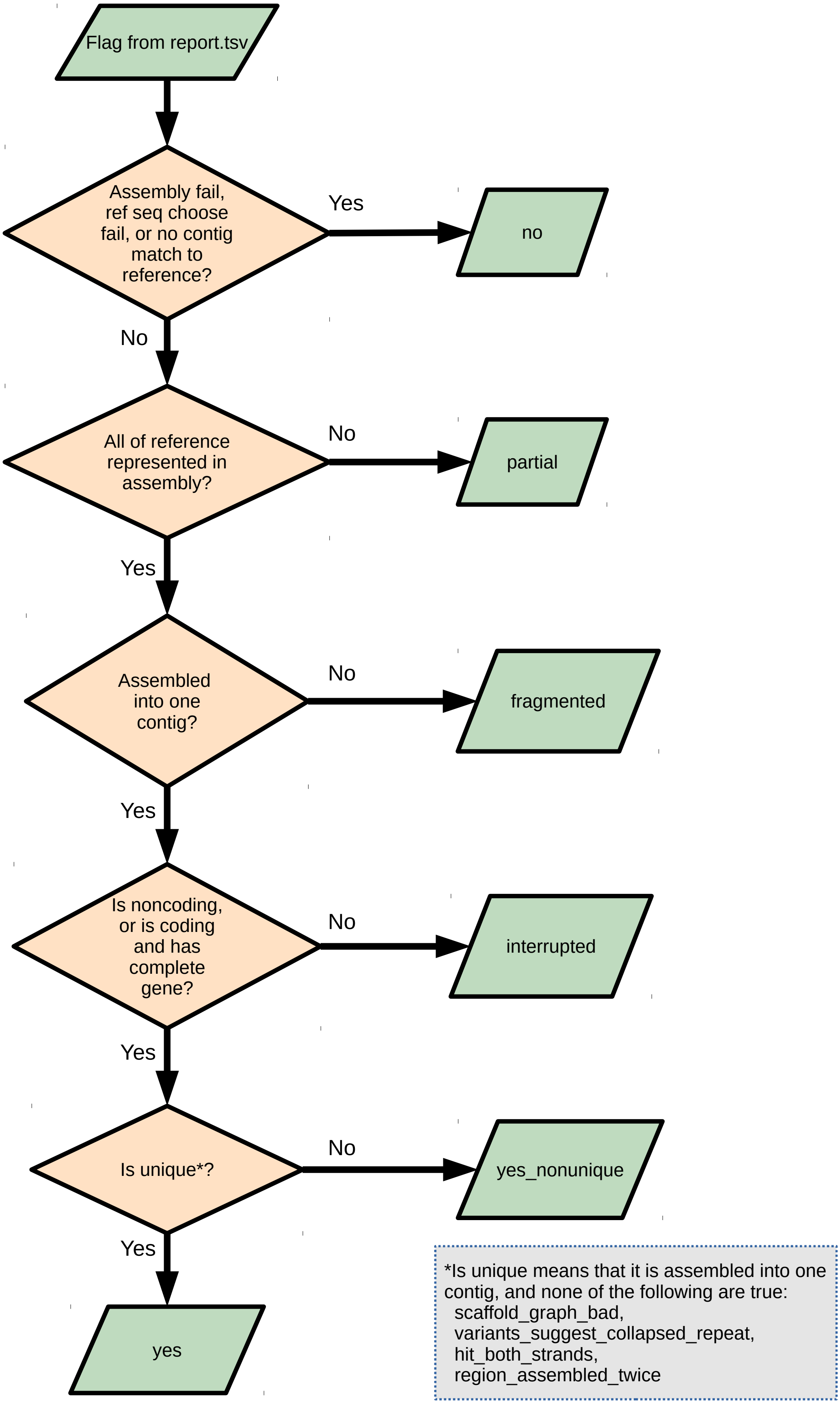

Figure S4: Method used for calculating the 'assembled' column output by `ariba summary`

3 *E. faecium*

The SRST2 version of the ARG-ANNOT sequences were downloaded and formatted for ARIBA with the following command.

```
ariba getref srst2_argannot ariba_db.download
```

The vanS-B gene, called "47__VanS-B_Gly__VanS-B__1672 no;yes;VanS-B;Gly;AY655721; 731-2073;1343" by SRST2, originally from ARG-ANNOT, was missing its final nucleotide A. This was confirmed by comparing with the GenBank record AY655721. It would cause ARIBA to exclude this sequence because the translation into amino acids results in a sequence that does not end with a stop codon. Therefore an A was added to the end of the sequence (called "VanS-B_Gly.47__VanS-B_Gly__VanS-B__1672") in the FASTA file `ariba_db.download.fa`. The data were then prepared for running the ARIBA pipeline with the command

```
ariba prepareref -f ariba_db.download.fa \
-m ariba_db.download.tsv ariba_db
```

and the ARIBA pipeline was run on each sample with

```
ariba run ariba_db reads_1.fq.gz reads_2.fq.gz ariba.out
```

For KmerResistance, we used the original file `ARGannot.r1.fasta` from the SRST2 github repository. The template database was made by running

```
maketemplatedb.py -i ARGannot.r1.fasta -o ARGannot.r1.fasta.kres_db
```

and KmerResistance was run on each sample with the command

```
zcat reads_1.fq.gz reads_2.fz.gz | KmerResistance.py \
-t_db ARGannot.r1.fasta.kres_db -o out1 -o2 out2 -w \
-s_db kmerresistance/database/complete_genomes.ATGAC
```

where `complete_genomes.ATGAC` is the prefix of files that are included with KmerResistance. SRST2 was run using the defaults options using the same reference file as KmerResistance. The command run on each sample was:

```
srst2 --input_pe reads_1.fq.gz reads_2.fq.gz --output out \
--log --gene_db ARGannot.r1.fasta
```

3.1 MLST calling

For MLST calling, the ARIBA reference data was downloaded from PubMLST and formatted for use with ARIBA using the command

```
ariba pubmlstget 'Enterococcus faecium' ariba_db.mlst
```

and ARIBA was run on each sample with

```
ariba run ariba_db.mlst/ref_db reads_1.fq.gz reads_2.fq.gz ariba.out
```

The reference data was downloaded for SRST2 using the command

```
getmlst.py --species "Enterococcus faecium"
```

where `getmlst.py` is the script included with SRST2, and then SRST2 was run on each sample with the command

```
srst2 --input_pe reads_1.fq.gz reads_2.fq.gz --output out \
--log --mlst_db Enterococcus_faecium.fasta \
--mlst_definitions srst2_pubmlst/efaecium.txt \
--mlst_delimiter '_'
```

3.2 Investigation of 7 genes in VanB operon

Only the VanB cluster had more than one gene. All tools gave the same reference sequence (where the gene was present), except for SRR980582, where ARIBA and SRST2 chose `VanB_1058` and KmerResistance chose `VanB_1060`.

All other differences were in the presence/absence of genes. Several of the differences were in samples genotyped to be VSE (see Supplemetary Table S1), where the differences appear to be due to marginal calls from low level contamination. The remaining differences, discussed below, were all in VRE samples.

All tools agreed for *vanB*, *vanH*, *vanR*, *vanS*, and *vanX*, the differences were all in *vanW* and *vanY*.

***vanW*.** Samples SRR980557, SRR980566, SRR980567, SRR980576, SRR980580, SRR980581, and SRR9805803 were called by ARIBA as having nonsense mutations, and SRR980574 with a frameshift. SRST2 called all these samples as "VanW-B_487*?", except for SRR980557 which was called as "VanW-B_487*" suggesting that it is present in the sample. KmerResistance reported all these samples as having the *vanW* gene. Further, ARIBA reported that the seven nonsense mutations were identical, changing the amino acid W to a stop codon at nucleotide position 571 in the reference gene. This was confirmed by running the following command on each BAM file output by SRST2:

```
samtools mpileup -L 100000 -t INFO/AD -A -f ARGannot.r1.fasta \
 -u -v -r 239__VanW-B_Gly__VanW-B__487:571-573 in.bam
```

where it was clear from the output that the codon TGG in the reference gene was changed to TGA in each sample. For example, the following lines are output from sample SRR980583

```
239__VanW-B_Gly__VanW-B__487 571 . T <*>   0 . DP=612;AD=591,0;
239__VanW-B_Gly__VanW-B__487 572 . G <*>   0 . DP=611;AD=585,0;
239__VanW-B_Gly__VanW-B__487 573 . G A,<*> 0 . DP=589;AD=0,564,0;
```

where each line has been truncated to fit on the page, with just the relevant information shown.

***vanY* in sample SRR980559.** This was called by ARIBA and SRST2, but not by KmerResistance. ARIBA reported 17 nonsynonymous amino acid changes, and SRST2 reported "44snp1indel". Given that ARIBA reported 94.42% identity between its assembly and the reference gene, we assume that the sample was too distant to be identified by KmerResistance.

***vanY* in sample SRR980565.** This was called by KmerResistance, with 95% coverage, but not by ARIBA or SRST2. ARIBA produced an assembly of 670 of the 807bp gene at 100% identity, therefore reporting the gene as not present. This was confirmed upon viewing the BAM file produced by SRST2, as shown in Supplementary Figure S7.

***vanY* in sample SRR980581.** ARIBA called this as "interrupted" because of a frameshift. SRST2 reported "VanY-B_1502*", with "1indel", and KmerResistance reported it as present, with 100% coverage of the gene.

The frameshift was verified by running the folloing command on the BAM file made by SRST2

```
samtools mpileup -L 100000 -t INFO/AD -A \
 -f ARGannot.r1.fasta -u -v \
 -r 262__VanY-B_Gly__VanY-B__1502 out__reads.ARGannot.r1.sorted.bam \
 | bcftools call -c -v -
```

which reported this:

```
262__VanY-B_Gly__VanY-B__1502 122 . TGGGG TGGGGG 214.458 .
  INDEL;IDV=974;IMF=0.879855;DP=1107;AD=1,795;VDB=0.0048673;SGB=-0.693147;
  MQSB=1;MQ0F=0;AF1=1;AC1=2;DP4=0,1,434,361;MQ=20;FQ=-289.528;
  PV4=0.454774,1,1,0.240182   GT:PL   1/1:255,255,0
```

ie a one base insertion in the reads, supported at high quality by 795 of the 796 reads mapped to that location.

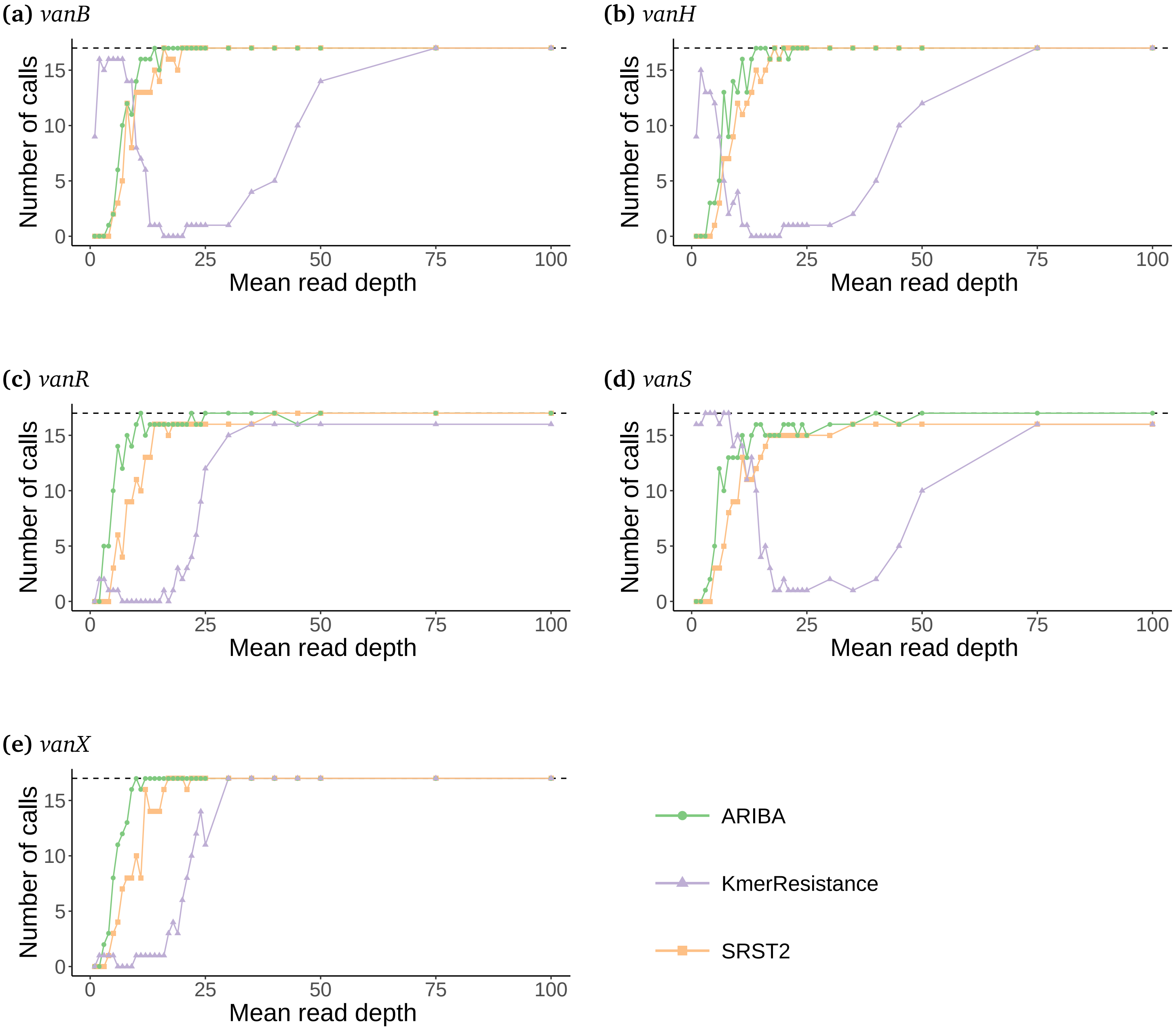

Figure S5: Effect of read depth on calling genes on *E. faecium* dataset.

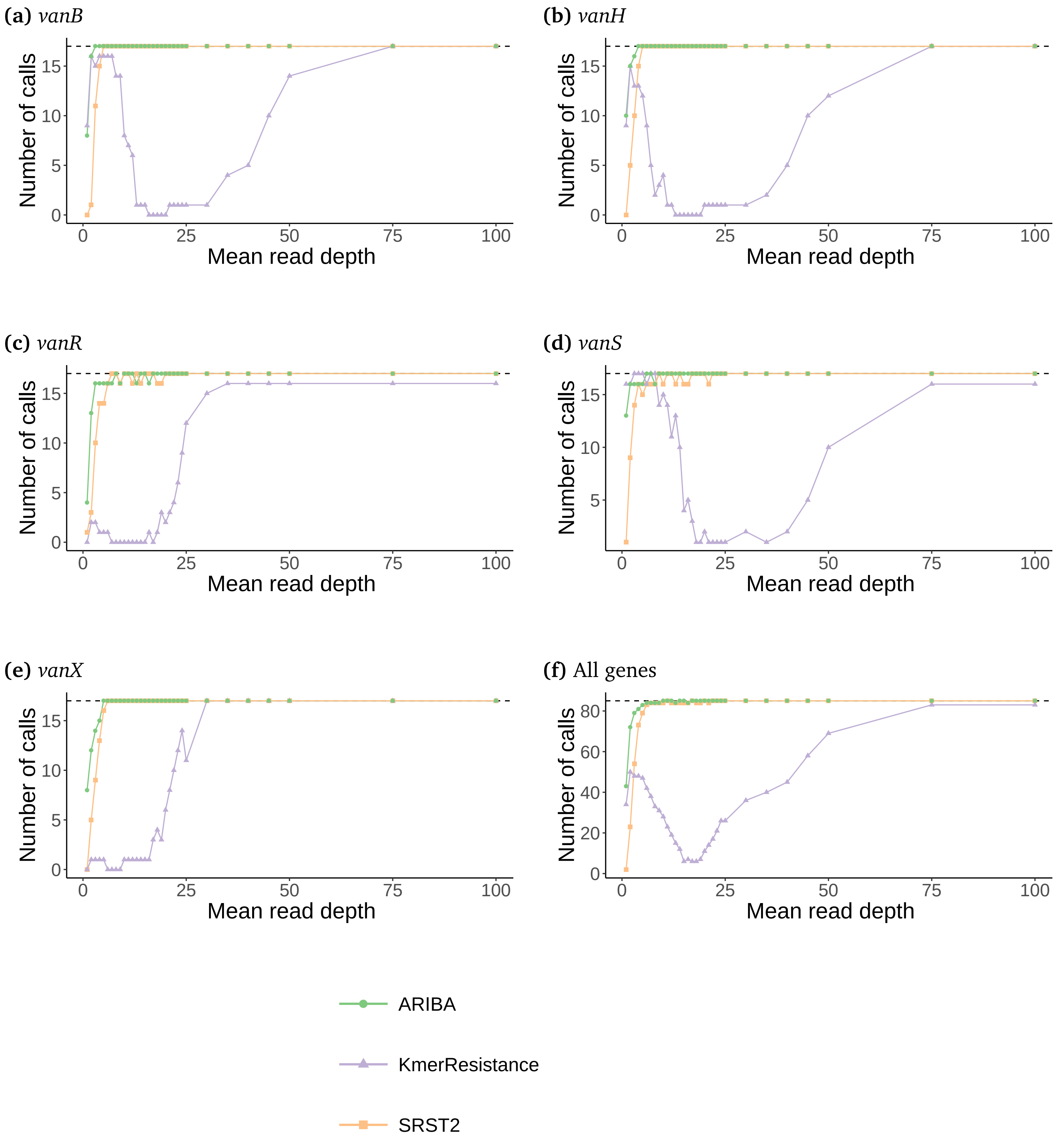

Figure S6: Effect of read depth on calling genes on *E. faecium* dataset, with more permissive criteria than those used in the main manuscript and in Supplementary Figure S5. Here, we additionally include calls made by SRST2 with a "?", and calls made by ARIBA where the assembly is identified as partial, fragmented, or interrupted.

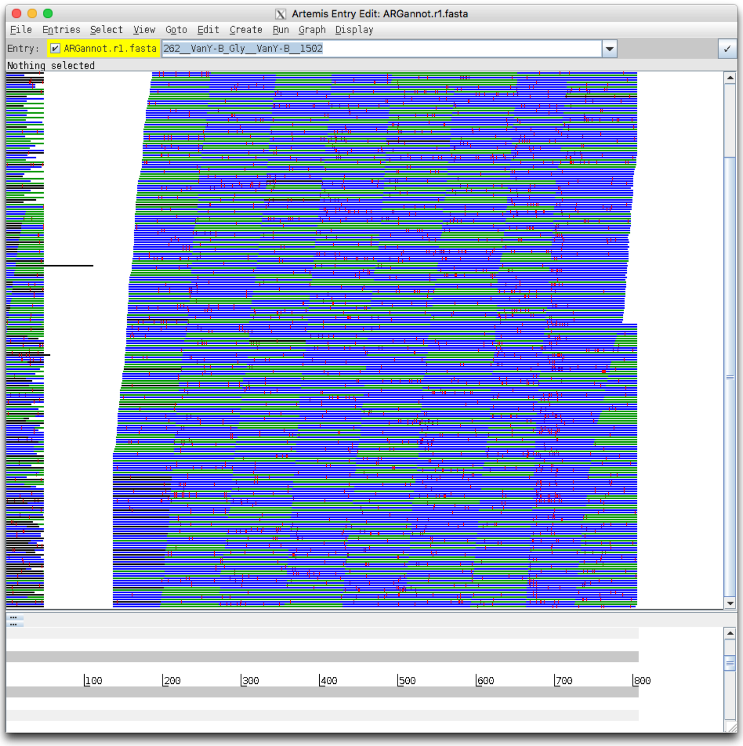

Figure S7: Artemis screenshot showing reads from sample SRR980565 mapped to the *vanY* gene.

4 *S. sonnei*

4.1 Reference data

The extra reference sequences, the details of which are in Supplementary Table S3, were downloaded using the script `s_sonnei_get_extra_ref_seqs.py` (which is included in the `Scripts/` directory of the ariba-publication GitHub repository). The CARD data was downloaded with the command

```
ariba getref --version 1.1.2 card card.getref
```

and prepared to run with ARIBA by running

```
ariba prepareref -f card.getref.fa -m card.getref.tsv \
 -f ref_data.sequences.fa -m ref_data.metadata.tsv ariba_db
```

The reference sequences were written to a FASTA file, `srst2.fa`, compatible for use with SRST2 by running

```
make_srst2_fa.py ariba_db srst2.fa
```

Finally, the data were prepared for use with KmerResistance using the two commands

```
sed 's/_/-/g' ariba_db/02.cdhit.all.fa > kres_db.input.fa

maketemplatedb.py -i kres_db.input.fa -o kres_db
```

A comparison of read depth of genes called by ARIBA and KmerResistance shown in Supplementary Figure S8.

A comparison of ARIBA local assembly and reference *strA* gene for sample ERR024606 shown in Supplementary Figure S10, and for *strB* and ERR024606 in Supplementary Figure S11.

4.2 Verify *gyrA* SNPs

The following command was used on the SRST2 BAM file for samples ERR028676 and ERR028677 to verify the presence or absence of one of the SNPs S83L, D87G, or D87Y:

```
samtools mpileup -L 100000 -t INFO/AD -A -f srst2.fa -uv \
 -r 569__gyrA-7__gyrA.3003294.U00096.2336792-2339420.2052__1821:$start-$end \
 in.bam
```

where `$start-$end` took the values `247-249` and `259-261`. The output for sample ERR028676 was

```
569__gyrA-7__gyrA.3003294.U00096.2336792-2339420.2052__1821 247 . T <*> 0
  . DP=170;AD=168,0;
569__gyrA-7__gyrA.3003294.U00096.2336792-2339420.2052__1821 248 . C <*> 0
  . DP=170;AD=169,0;
569__gyrA-7__gyrA.3003294.U00096.2336792-2339420.2052__1821 249 . G <*> 0
  . DP=171;AD=171,0;
569__gyrA-7__gyrA.3003294.U00096.2336792-2339420.2052__1821 259 . G <*> 0
  . DP=172;AD=167,0;
569__gyrA-7__gyrA.3003294.U00096.2336792-2339420.2052__1821 260 . A <*> 0
  . DP=173;AD=173,0;
569__gyrA-7__gyrA.3003294.U00096.2336792-2339420.2052__1821 261 . C <*> 0
  . DP=173;AD=173,0;
```

confirming that none of the SNPs of interest were present. The output for sample ERR028677 was

```
569__gyrA-7__gyrA.3003294.U00096.2336792-2339420.2052__1821 247 . T <*> 0
  . DP=31;AD=31,0;
569__gyrA-7__gyrA.3003294.U00096.2336792-2339420.2052__1821 248 . C T,<*> 0
  . DP=30;AD=0,30,0;
569__gyrA-7__gyrA.3003294.U00096.2336792-2339420.2052__1821 249 . G <*> 0
  . DP=30;AD=30,0;
569__gyrA-7__gyrA.3003294.U00096.2336792-2339420.2052__1821 259 . G <*> 0
  . DP=30;AD=29,0;
569__gyrA-7__gyrA.3003294.U00096.2336792-2339420.2052__1821 260 . A <*> 0
  . DP=30;AD=29,0;
569__gyrA-7__gyrA.3003294.U00096.2336792-2339420.2052__1821 261 . C <*> 0
  . DP=31;AD=30,0;
```

confirming the SNP S83L (a codon change from TCG to TTG).

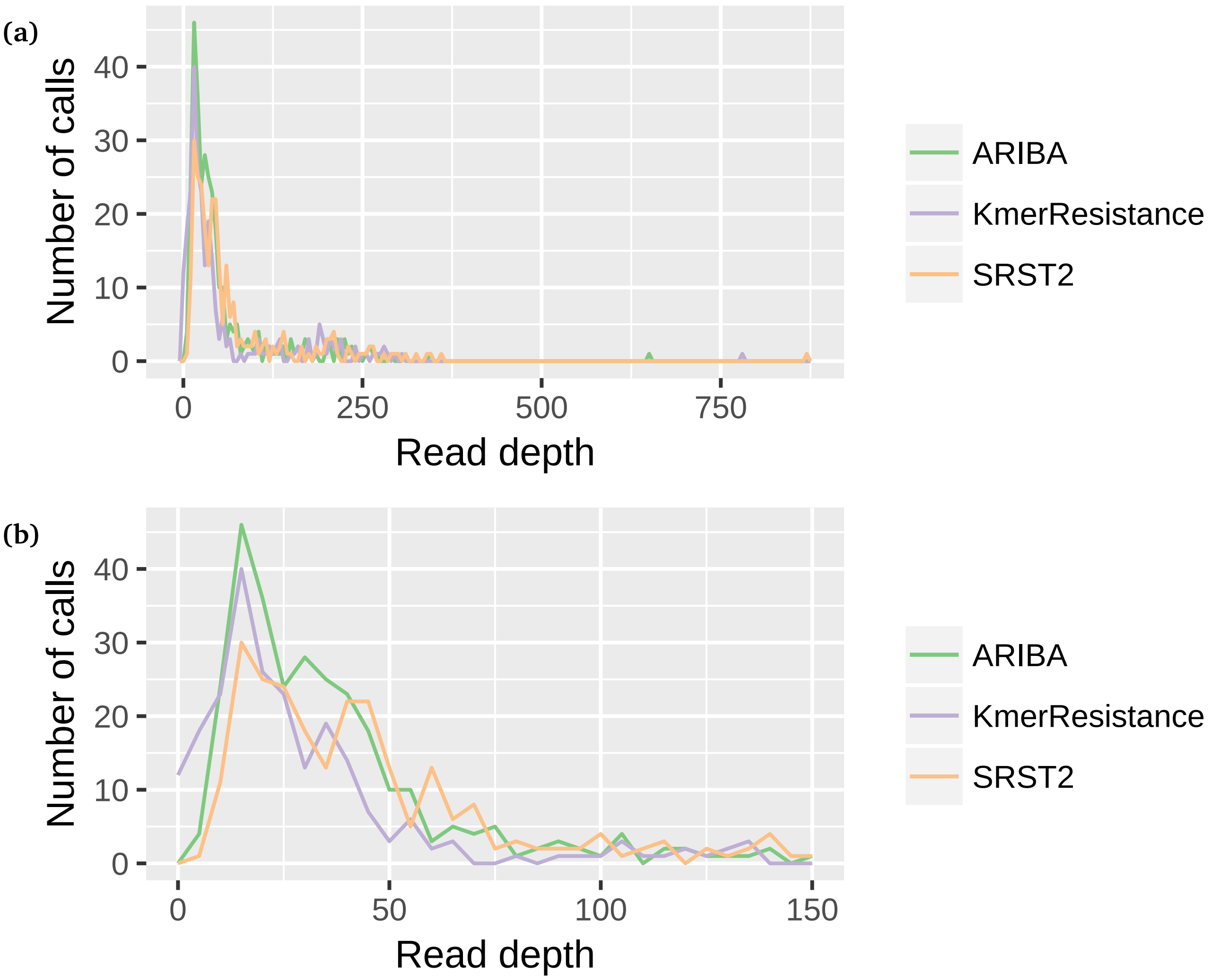

Figure S8: Comparison of read depth of genes called by ARIBA, KmerResistance and SRST2 on the *S. sonnei* data set. (a) shows all values of reported read depths, whereas (b) shows only the range 0–150, so that the details are visible at low depth.

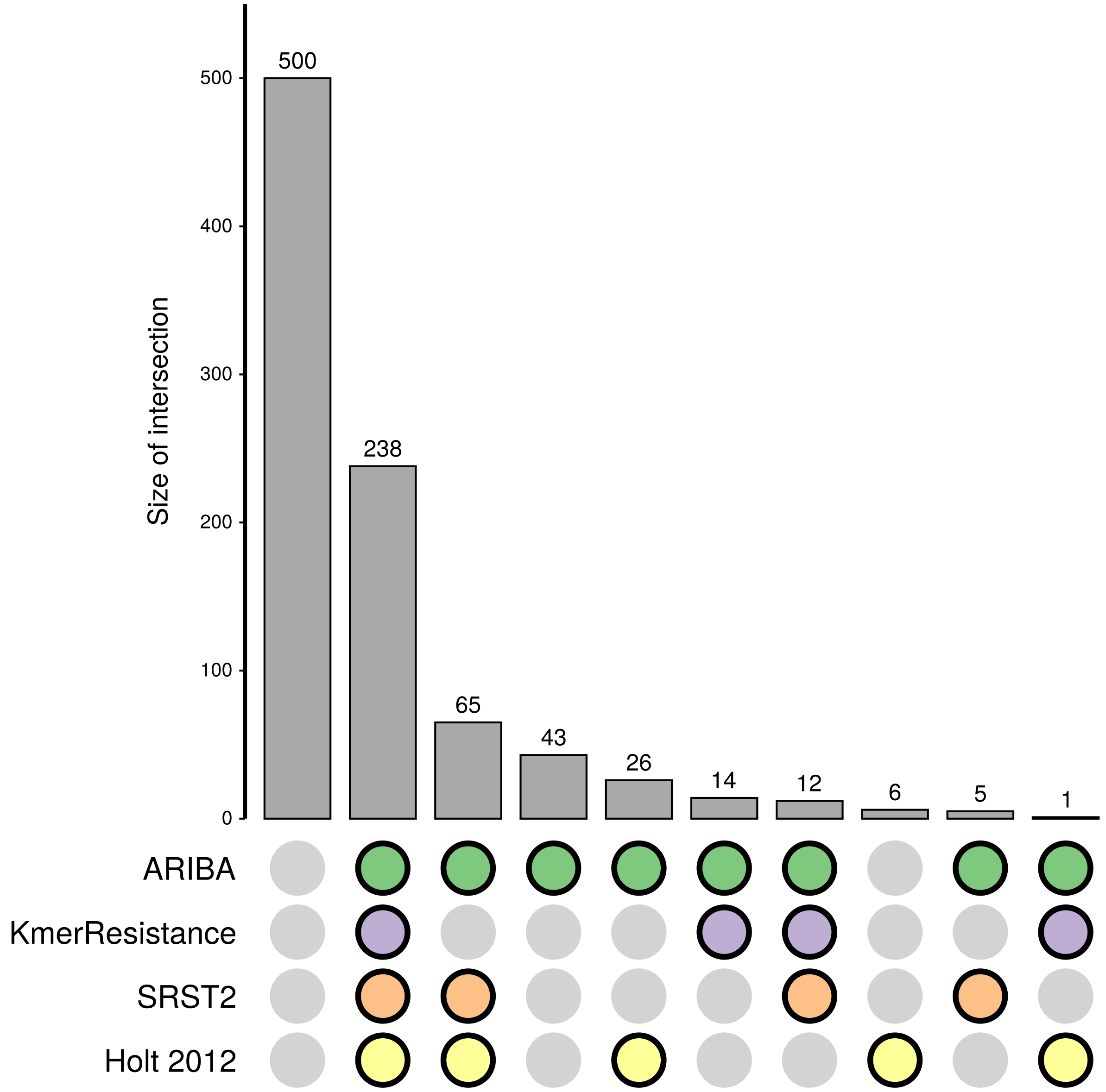

Figure S9: Concordance between AMR calling methods on the *S. sonnei* data. A coloured dot indicates which methods were in agreement. The first column illustrates where no resistance mechanisms were predicted. This is generated using more permissive rules than in Figure 3. Here, we additionally include calls made by SRST2 with a "?", and calls made by ARIBA where the assembly is identified as partial, fragmented, or interrupted.

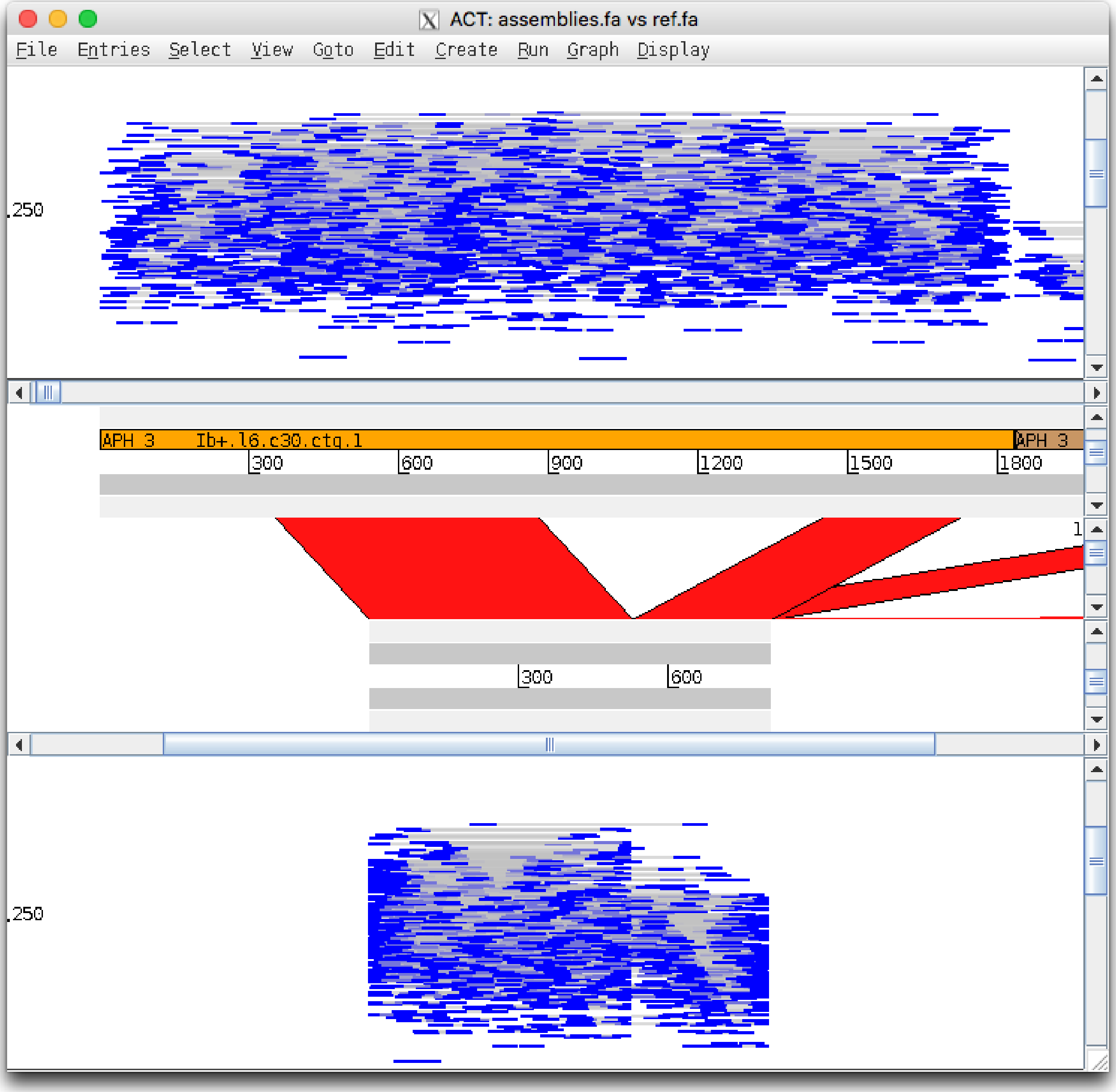

Figure S10: Sample ERR024606 *strA* gene. ACT screenshot showing a comparison between the ARIBA assembly (top) of *strA* and the reference sequence (bottom). Nucmer matches between the sequences are shown in red. The mapped reads are shown in "inferred size" view, where reads are shown in blue, with each pair connected with a grey line. The height of each read pair is determined by the inferred insert size from the mapping. The BAM file made by SRST2 was used for the reference sequence, and the reads were mapped to the ARIBA contigs using Bowtie2 with the default settings. The position of the insertion is clear in the reads mapped to the reference sequence, whereas there is consistent coverage across the ARIBA assembly.

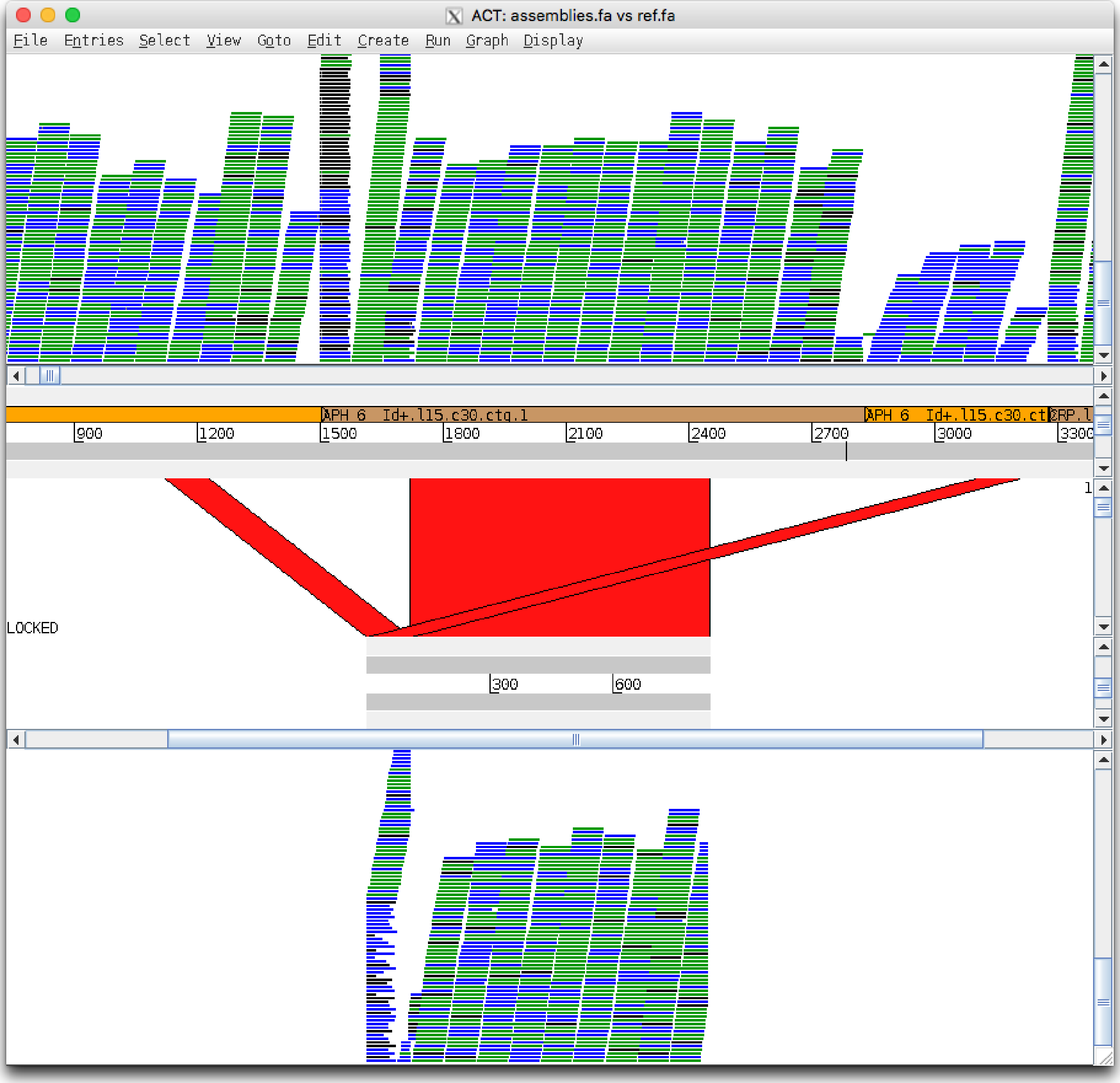

Figure S11: Sample ERR028673 *strB* gene, showing a comparison of the ARIBA assembly (top) and the reference sequence (bottom) together with the mapped reads. Reads were mapped in the same way as explained for Supplementary Figure S10.

5 *N. gonorrhoeae*

The reference data were prepared for ARIBA using the methods described in the main text. The workflow is automated in the script `n_gonorrhoeae_make_ariba_db.sh`, which contains all the commands that were used and is included in the `Scripts/` directory of the ariba-publication GitHub repository.

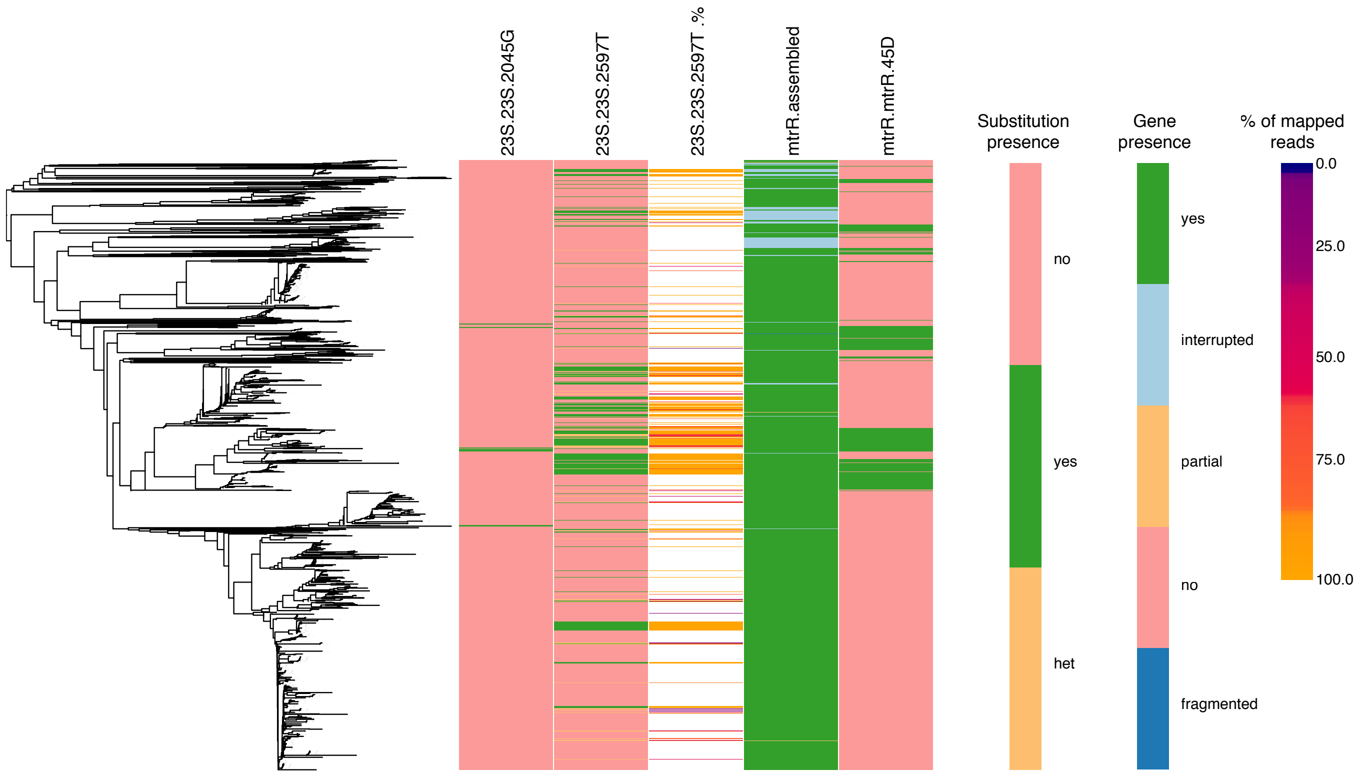

Figure S12: Phandango visualisation of ARIBA results for *N. gonorrhoeae* aligned against a phylogenetic tree of the isolates to illustrate that resistance determinants have emerged multiple times in the population. Colours in columns showing the presence (yes) or absence (no) of a particular substitution are indicated in the substitution presence key. For the 23S C2597T substitution the percentage of mapped reads containing the substitution are shown in the 2597T.% column, for which the key is labelled % of mapped reads. The meaning of colours in the mtrR.assembled column are shown in the gene presence key, and indicate isolates for which the assembly for the *mtrR* gene is complete (yes), interrupted, partial, fragmented or absent (no).

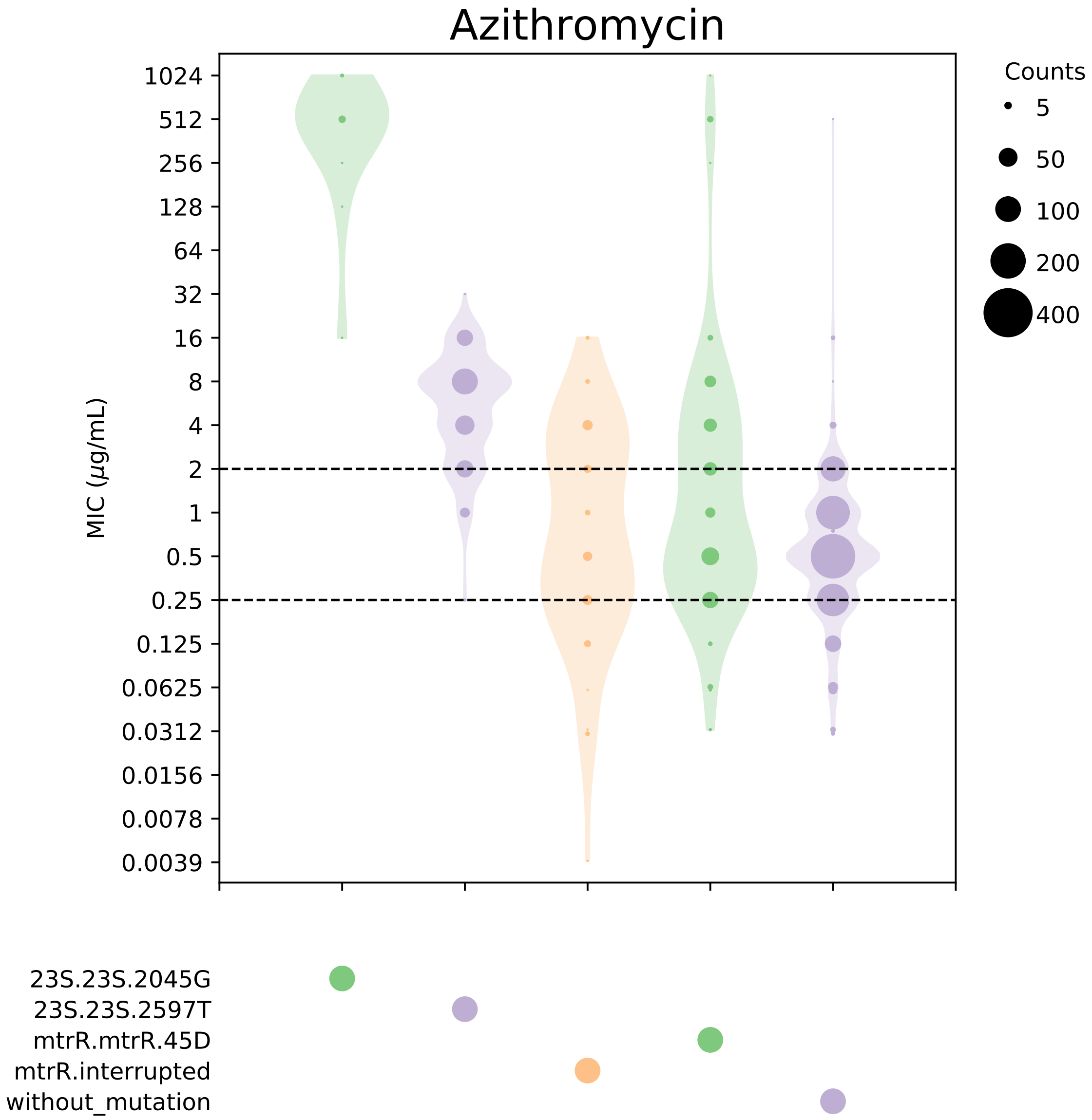

Figure S13: Distribution of MICs (represented on a logarithmic scale) for azithromycin for all relevant AMR determinants in our custom database. Dotted horizontal lines mark clinical breakpoints as described in Figure 4.

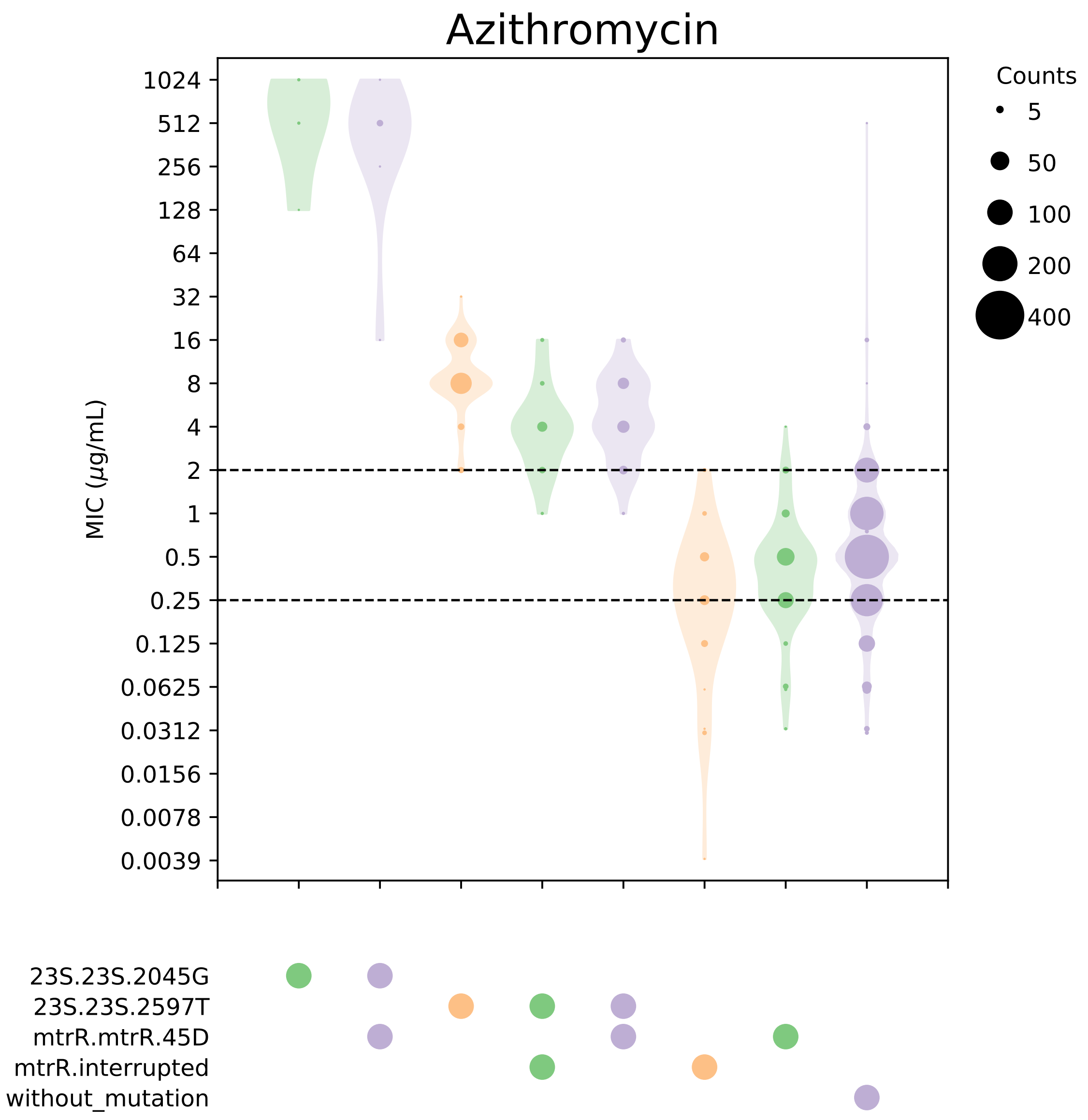

Figure S14: Distribution of MICs (represented on a logarithmic scale) for azithromycin for all observed combinations of relevant AMR determinants in our custom database excluding heterozygous hits. Dotted horizontal lines mark clinical breakpoints as described in Figure 4.

6 Run time and memory

Box plots of the run time and memory are shown in Supplementary Figure S15 for the *E. faecium* and *S. sonnei* data sets. The values used for wall clock time and peak memory usage were those reported from using the UNIX command `time -v`. Specifically, wall clock time is taken from the value of "Elapsed (wall clock) time" and peak memory from the value of "Maximum resident set size". The raw data are given in Supplementary Table S7, and original output from `time -v` is included in the GitHub repository.

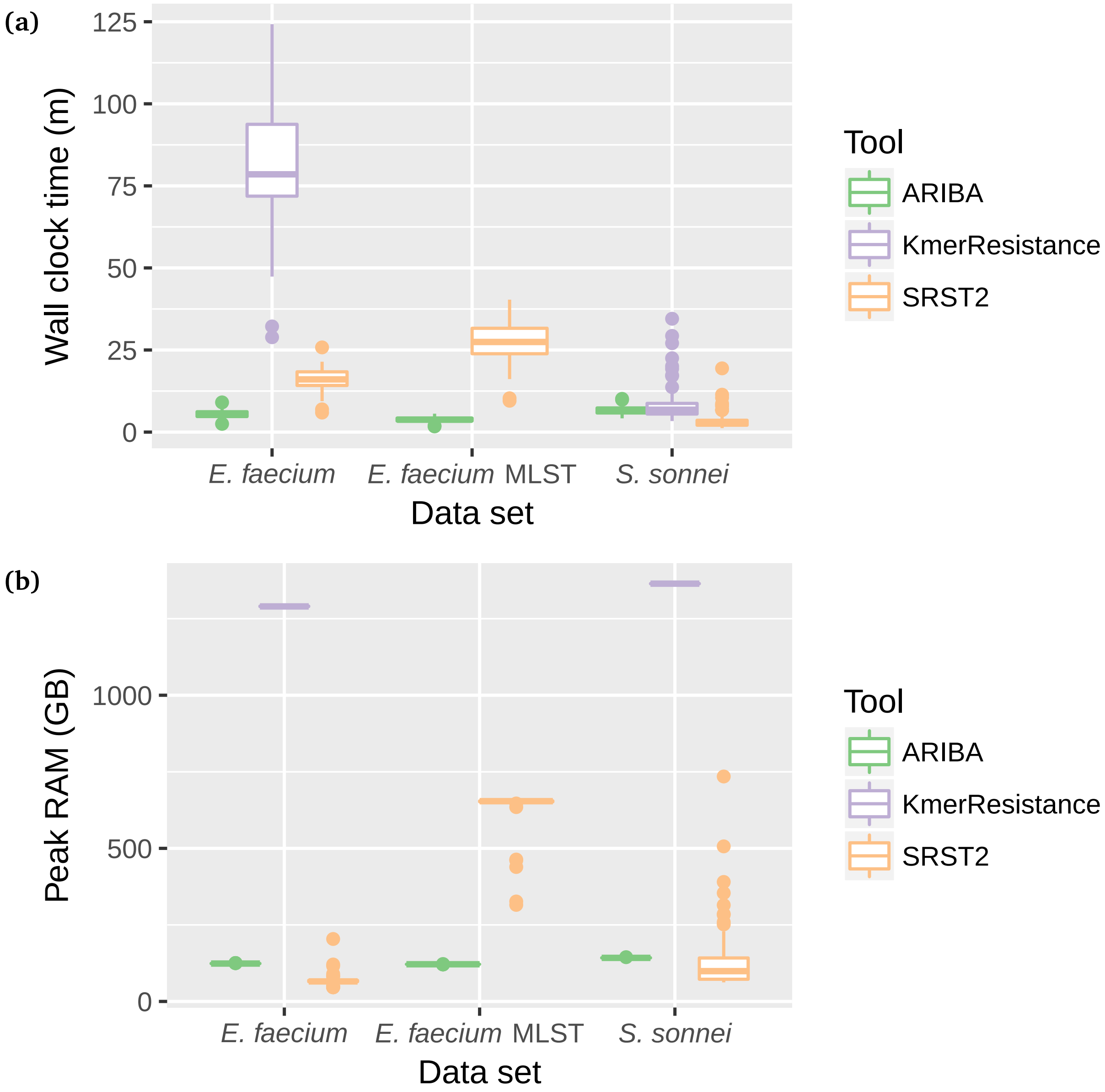

Figure S15: (a) Run time and (b) memory usage on the *E. faecium* and *S. sonnei* datsets.
